# No action is without its side effects: Adverse drug reactions and missed doses of antituberculosis therapy, a scoping review

**DOI:** 10.1111/bcp.15908

**Published:** 2023-10-09

**Authors:** Eleanor G. Dixon, Shaista Rasool, Brian Otaalo, Ashmika Motee, James W. Dear, Derek Sloan, Helen R. Stagg

**Affiliations:** ^1^ Usher Institute University of Edinburgh Edinburgh UK; ^2^ Infectious Disease Institute, Makerere University College of Health Sciences Kampala Uganda; ^3^ NIHR RIGHT4 Centre for Poisoning, Centre for Cardiovascular Science University of Edinburgh Edinburgh UK; ^4^ School of Medicine University of St Andrews St Andrews UK; ^5^ Department of Infectious Disease Epidemiology London School of Hygiene & Tropical Medicine London UK

**Keywords:** drug‐related side effects and adverse reactions, lost to follow‐up, review, treatment adherence and compliance, tuberculosis

## Abstract

**Aims:**

A key reason for the failure of antituberculosis (anti‐TB) treatment is missed doses (instances where medication is not taken). Adverse drug reactions (ADRs) are 1 cause of missed doses, but the global evidence, their relative contribution to missed doses *vs*. other causes, the patterns of missed doses due to ADRs and the specific ADRs associated with missed doses have not been appraised. We sought to address these questions through a scoping review.

**Methods:**

MEDLINE, Embase and Web of Science were searched on 3 November 2021 using terms around active TB, missed doses and treatment challenges. Studies reporting both ADR and missed dose data were examined (PROSPERO: CRD42022295209).

**Results:**

Searches identified 108 eligible studies: 88/108 (81%) studies associated ADRs with an increase in missed doses; 33/61 (54%) studies documenting the reasons for missed doses gave ADRs as a primary reason. No studies examined patterns of missed doses due to ADRs; 41/108 (38%) studies examined associations between 68 types of ADR (across 15 organ systems) and missed doses. Nuance around ADR‐missed doses relations regarding drug susceptibility testing profile and whether the missed doses originated from the patient, healthcare professionals, or both were found.

**Conclusion:**

There is extensive evidence that ADRs are a key driver for missed doses of anti‐TB treatment. Some papers examined specific ADRs and none evaluated the patterns of missed doses due to ADRs, demonstrating a knowledge deficit. Knowing why doses both are and are not missed is essential in providing targeted interventions to improve treatment outcomes.

**What is already known about this subject**

- Adverse drug reactions (ADRs) are a known risk factor for missed doses of antituberculosis therapy.
- Relationships between different missed dose patterns and treatment outcomes are poorly understood.

**What this study adds**

- ADRs are a primary driver of missed doses.
- Papers examining which patterns of missed doses are due to ADRs are not present in the literature.
- ADRs of antituberculosis therapy affect many organ systems and have a complex relationship with missed doses; nuance around drug susceptibility testing profiles and whether the missed doses originated from the patient, healthcare professionals, or both must be considered when designing interventions.

## 1 INTRODUCTION

Over the last decade, tuberculosis (TB) has killed more individuals than any other infectious disease, an estimated 1.6 million people per year worldwide.^1^ However, between 2000 and 2021, 74 million lives have been saved through anti‐TB treatment.^2^

Missed doses (i.e. instances where medication is not taken) of anti‐TB therapy are thought to be 1 of the primary reasons for unfavourable outcomes from anti‐TB treatment.^3^ Not only do missed doses compromise the therapeutic capability of treatment regimens but they contribute to the development of drug resistance during treatment.^1^ As such, the World Health Organization (WHO) recommended that patients be observed taking every single dose of their anti‐TB medication by a healthcare professional (HCP); this is directly observed therapy.^4^ Directly observed therapy and digital adherence technologies have been used with varied success.^4^, ^5^, ^6^

Although it is widely accepted that missed doses are problematic, patterns of missed doses are unknown, and relationships between different missed‐dosing patterns and treatment outcomes are poorly understood. For example, a patient who sporadically misses doses poses different challenges pharmacologically and clinically to a patient who stops treatment early.^7^

HCPs, programme managers and researchers also need to know why people miss doses in order to build effective interventions and personalize treatment adherence support.

Adverse drug reactions (ADRs) from anti‐TB therapy are a known reason for missed doses.^8^ Common ADRs of first‐line anti‐TB treatment range widely in organ system affected and severity.^9^ The balance of drug toxicity relative to therapeutic gain changes for second‐line therapy, with toxicity and treatment duration increasing.^10^ Very little is known about which types of ADR are most likely to result in missed doses or the patterns of missed doses which commonly arise from each type of ADR. Analysis of the relationships between ADRs and missed doses is necessary to improve individualized patient care and TB treatment outcomes.

Given current international interest in missed doses of anti‐TB treatment and the importance of understanding their causes, we undertook a scoping review to:

- document the global evidence of an association between ADRs and missed doses;
- examine the relative contribution of ADRs as a cause of missed doses;
- describe the patterns of missed doses associated with ADRs; and
- analyse relationships between different types of ADRs and missed doses.

## 2 METHODS

Scoping reviews facilitate the identification of knowledge gaps, clarify concepts and explore the breadth of literature for set topics.^11^ We chose a scoping review methodology for this research to provide a broad overview of relationships between ADRs and missed doses of anti‐TB therapy, and highlight evidence gaps.^12^

### 2.1 Eligibility criteria

Eligible studies contained patients diagnosed with active TB, who were taking anti‐TB medication, and where relationships between ADRs and missed doses were described. Missed doses were defined as instances where ≥1 dose of anti‐TB therapy was missed. For a dose to have been counted as missed, all drugs needed to have not been taken; single drug or partial regimen changes were not included. This was due to the lack of, or variable reporting of single drug changes across the studies. Studies of both drug‐sensitive (DS) and all forms of drug‐resistant (DR) disease were included. All primary research studies were eligible, excluding case reports containing a single patient. Human studies in any language and with any publication date were included. Full eligibility criteria are detailed in Table S1.

### 2.2 Search strategy

On 3 November 2021, MEDLINE, Embase (both via Ovid) and Web of Science were searched using Medical Subject Headings (MeSH) and free text subject headings covering TB, missed doses and treatment challenges (Tables S2–S4). The development of the search terms is documented in Text S1.

#### 2.2.1 Screening

Hits were downloaded into Endnote 20 before being automatically and manually deduplicated. Deduplicated hits were uploaded to Covidence for screening. Titles and abstracts were screened, followed by the full texts of the remaining studies. Screening occurred in duplicate and was conducted by Eleanor Dixon (EGD) plus 1 of 3‐s reviewers (S.R., B.O. or A.M.). Eligible studies were finalized, and any disagreements were independently resolved by H.R.S. Review articles were identified during this process and their reference lists screened for eligible studies.

### 2.3 Data extraction

Data were extracted (22 fields) into predesigned forms in Covidence and Microsoft Excel; fields included demographic and clinical factors such as country of study and method of missed dose assessment. The association between ADRs and missed doses was recorded on a study level and a specific ADR level. The presence of each ADR was counted once per study (subsequently referred to as *occasions*). Texts S2 and S3 provide further details regarding data extraction.

### 2.4 Data synthesis

Data synthesis followed recommendations from other methodological papers, in particular Loke *et al*.'s publication on the reporting of systematic reviews of adverse effects.^13^

Acknowledging that decisions to omit anti‐TB treatment are sometimes made by patients, and sometimes prompted by HCP advice, studies were categorized for all analyses into those which reported patient‐originated, HCP‐originated, or patient‐and‐HCP‐originated missed doses. If the originator of the missed doses was not stated, the study design and methods were used to determine this. The HCP‐originated grouping included studies which reported temporary or permanent suspension of anti‐TB medication by HCPs (e.g., as part of clinical management for ADRs).

As different drug susceptibility testing (DST) profiles require treatment with different drug regimens that carry different toxicity risks, studies were also categorized by the DST profile of the assessed population: DS‐TB, DR‐TB or populations with mixed DST profiles. For this review, DR‐TB encompasses single drug‐resistant TB, multidrug‐resistant TB, and extensively drug‐resistant TB, according to WHO definitions at the time of study conduct.

Associations between ADRs and missed doses were interpreted for each study from statements in the manuscript text or tabulation of data. Associations were classified into 4 groups: (i) studies that found ADRs to be associated with an increase in missed doses; (ii) studies that found ADRs not to be associated with an increase in missed doses; (iii) studies that found mixed evidence of a relationship between ADRs and missed doses (i.e., some analyses within the same study provided evidence of an association with ADRs whilst others did not); and (iv) studies that found ADRs to be associated with a decrease in missed doses.

The relative contribution of ADRs as a cause of missed doses was also extracted for each study from statements in the manuscript text or data tables (Text S3). Examples of the former include where ADRs were described as a *primary* or *main* reason for missed doses. Where data describing causes of missed doses were tabulated, ADRs were classified as a primary reason when listed as the first or second most common cause.

The language used to describe ADRs identified in the context of missed doses was standardized. Medical Dictionary for Regulatory Activities (MedDRA) terminology^14^ was assigned to each reported ADR term to ensure a universal understanding of the ADR (Table S5). Where the intention of the ADR term was ambiguous, EGD and James Dear (JWD) independently assigned MedDRA terms before collectively confirming the terms. ADR terms were subsequently categorized by class organ systems (COS). ADRs categorized under multiple MedDRA COS were assigned to a single COS based upon ADR symptoms and treatment targets. This process was conducted independently by 2 authors (E.G.D. and J.W.D.).

### 2.5 Registration

PROSPERO CRD42022295209.

## 3 RESULTS

**FIGURE 1:**
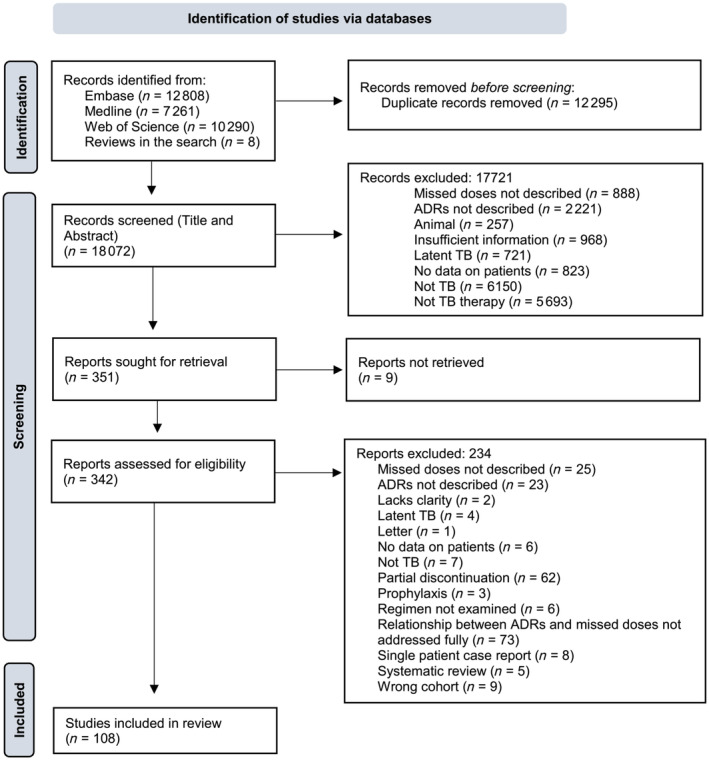
PRISMA flow chart. A chart showing the identification of eligible studies during the review. ADRs, adverse drug reactions; *n*, number of studies; TB, tuberculosis. Records were excluded for many reasons.

The search identified 30 359 hits. In total, 108 eligible studies were identified (Figure 1), with a mixture of sample sizes (5–7505), quantitative and qualitative data, and both prospective and retrospective designs. Data were available from ≥45 countries covering all 6 WHO global regions and from both adult and paediatric populations.^15^ Studies had a mixture of inclusion criteria as to underlying comorbidities and TB DST profiles (Table S6).

### 3.1 Missed dose definitions

Fifty‐two of the 108 (48%) studies stated the type of missed doses they were describing; 17 different definitions (Table S7) were used. Two main types of missed doses were present: early treatment stop (early discontinuation) and other types of missed doses.

### 3.2 Evidence for an association between ADRs and missed doses

It was possible to examine whether ADRs were associated with missed doses in all 108 studies. Seventy‐seven out of the 108 (71%) studies provided data on patient‐originated missed doses, 28/108 (26%) on HCP‐originated missed doses, and 3/108 (3%) studies on a combination of both patient‐originated and HCP‐originated missed doses (Table 1). ADRs were associated with an increase in missed doses in 86% of studies where patient‐originated missed doses were assessed, compared to 60% of studies where HCP‐originated missed doses were assessed.

**TABLE 1:** Association between adverse drug reactions (ADRs) and missed doses.

Origin of missed dose	Association shown	DST profile			
		DS	DR	Mixed	Totals
Patient (*n* = 77)	ADRs associated with an increase in missed doses	17	16	33	66 (86%)
	ADRs not associated with an increase in missed doses	2	2	3	7 (9%)
	ADRs associated with a decrease in missed doses	0	1	2	3 (4%)
	Mixed evidence of an association between ADR and missed doses	0	0	1	1 (1%)
HCP (*n* = 28)	ADRs associated with an increase in missed doses	6	5	6	17 (60%)
	ADRs not associated with an increase in missed doses	4	6	0	10 (35%)
	ADRs associated with a decrease in missed doses	0	0	0	0 (0%)
	Mixed evidence of an association between ADR and missed doses	1	0	0	1 (4%)
Patient and HCP (*n* = 3)	ADRs associated with an increase in missed doses	3	0	0	3 (100%)

Abbreviations: DR, drug resistant; DS, drug susceptible; DST, drug susceptibility testing; HCP, healthcare professional; TB, tuberculosis.

*Note*: All eligible studies (*n* = 108) reported an association between ADRs and missed doses of antituberculosis therapy. Studies were categorized by missed dose originator (the party responsible for omitting antituberculosis therapy) and the DST profile of each study's population.

When the relationship between ADRs and missed doses was categorized based on the DST profile amongst the study population (Table 1), 26/33 (79%) DS‐TB‐specific studies reported that ADRs were associated with an increase in missed doses compared to 21/30 (70%) DR‐TB specific studies.

ADRs were not associated with an increase in missed doses in 17/108 (16%) studies, 7 of which provided data on patient‐originated missed doses and 10 of which, data on HCP‐originated missed doses. Data examining this association came from patients with a range of DST profiles (Table 1).

In 3 studies, ADRs were associated with a decrease in missed doses. All 3 studies assessed patient‐originated missed doses: 1 evaluated DR‐TB patients only, whilst the other 2 evaluated patients with mixed DST profiles. One of these studies was a prospective DS‐TB cohort study which reported patients' ADRs on an individual level and found that different patients had varying relationships between ADRs and missed doses.^16^ The other was a mixed‐methods study of a mixed DST profile population within which qualitative data drew an association between ADRs and increased missed doses whereas quantitative did not.^17^

### 3.3 The relative contribution of ADRs as a cause of missed doses

Sixty‐one/108 (56%) studies examined multiple potential causes of missed doses, enabling examination of the relative contribution of ADRs as a cause of missed doses compared to other causes.

Fifty‐five of these studies included patient‐originated missed doses and, of these, 31 (56%) identified ADRs as a primary cause. Six included HCP‐originated missed doses and, of these, 2 (33%) identified ADRs as a primary cause.

Examples of other causes of missed doses identified in the studies included: financial limitations, impact on self‐perception or stigma, change in social circumstance (e.g. moved further away from the treatment facility, changes in family responsibility) and being asymptomatic/feeling as though symptoms had improved.

### 3.4 Patterns of missed doses associated with ADRs

None of the studies reported these data for either patient‐ or HCP‐originated missed doses.

### 3.5 Association between specific types of ADRs and missed doses

Forty‐one of the 108 (38%) studies examined associations between 68 MedDRA‐classified types of ADR (Table S5) and missed doses. Specific associations were examined on 178 occasions in these studies (Table S8). The granularity of data between studies was variable: some ADRs were only reported at COS level (e.g. *haematological disorders*), whereas other studies reported more specific events (e.g. *anaemia* or *thrombocytopenia*). For consistency of cross‐study analysis, Table 2 summarizes this whole dataset at COS level. ADRs were assessed across 15 COS, with 69% of reports concentrated within 5 COS: *gastrointestinal disorders* (23%), *hepatobiliary disorders* (13%), *nervous system disorders* (12%), *skin and subcutaneous tissue disorders* (11%), and *psychiatric disorders* (10%). ADRs within these 5 COS were also the most commonly reported ADRs associated with an increased in missed doses.

**TABLE 2:** Studies containing evidence of an association between adverse drug reaction (ADR) type and missed doses.

Class organ system	Studies showing evidence of an association between ADR type and increased missed anti‐TB drug doses												Row totals *N*, (%)
	DS‐TB			DR‐TB			Mixed DST population			All studies			
	Yes	No	Mixed	Yes	No	Mixed	Yes	No	Mixed	Yes	No	Mixed	
Blood and lymphatic system disorders	6	2	1	0	0	0	1	0	0	7	2	1	10 (6)
Cardiac disorders	0	0	0	1	0	0	0	0	0	1	0	0	1 (<1)
Ear and labyrinth disorders	0	0	0	0	5	0	0	0	0	0	5	0	5 (3)
Endocrine disorders	0	0	0	0	2	0	0	0	0	0	2	0	2 (1)
Eye disorders	1	0	0	0	2	0	1	0	0	2	2	0	4 (2)
Gastrointestinal disorders	8	4	0	5	9	0	16	0	0	29	13	0	42 (24)
General disorders and administration site conditions	2	1	0	2	0	0	4	0	0	8	1	0	9 (5)
Hepatobiliary disorders	4	6	0	3	5	0	5	0	0	12	11	0	23 (13)
Immune system disorders	1	0	1	0	1	0	1	0	0	2	1	1	4 (2)
Metabolism and nutrition disorders	0	1	0	1	0	0	2	0	0	3	1	0	4 (2)
Musculoskeletal and connective tissue disorders	3	0	1	0	4	0	1	0	0	4	4	1	9 (5)
Nervous system disorders	5	3	1	3	5	0	5	0	0	13	8	1	22 (12)
Psychiatric disorders	4	0	0	5	5	0	3	0	0	12	5	0	17 (10)
Renal and urinary disorders	1	0	0	0	4	0	2	0	0	3	4	0	7 (4)
Skin and subcutaneous tissue disorders	7	0	2	0	4	0	6	0	0	13	4	2	19 (11)
Column total	42	17	6	20	46	0	47	0	0	109	63	6	178

*Note*: An assessment of the associations between types of ADRs from different MedDRA COS and an increase in missed doses of anti‐TB therapy. Where an ADR in a study was recorded as both associated with an increase and not associated with an increase in missed doses (often found in different patients), the association was described as mixed. The presence of each ADR was counted once per study (*n*). Studies were grouped by DST profiles of the study participants.

Out of the 178 occasions specific ADRs were reported in the context of missed doses, 109/178 (61%) occasions were associated with increased missed doses of anti‐TB therapy, 63/178 (36%) occasions were not associated with increased missed doses, and in 6 (3%) evidence was mixed. Associations between reported ADR type and increased missed doses were more frequent in studies of DS‐TB than DR‐TB (42/65 (65%) reports compared with 20/66 (30%), respectively).

At the COS level, all available evidence for *cardiovascular disorders* indicated that ADRs are associated with increased missed doses; however, the data were derived from a single study which evaluated bradycardia in DR‐TB patients. For 2 COS (*ear and labyrinth disorders* and *endocrine disorders*), the evidence was unanimous that ADRs were not associated with increased missed doses; data for these COS were derived from 5 studies of DR‐TB patients. For the remaining 12 COS, patterns were inconsistent; some studies reported an association between ADRs and increased missed doses whilst others did not. In 10 COS, the majority (>50%) of assessments showed an association with increased missed doses, and in only 1 (*renal and urinary disorders*) most assessments showed no association.

## 4 DISCUSSION

To our knowledge, this is the first review directly examining how individual ADRs impact the taking of anti‐TB therapy; 81% of included studies from around the world documented at least some evidence for an association between ADRs and an increase in missed doses. Critically, over half of the relevant studies comparing factors contributing to missed doses identified ADRs as a primary cause.

We found no studies that described the patterns of missing doses in response to ADRs, demonstrating a key knowledge gap. The current widely used threshold of *patient adherence* being 80–90% of therapy taken^7^ masks the complexity of dose taking during treatment—patients who sporadically miss individual doses of anti‐TB therapy pose different treatment challenges to patients who discontinue therapy early. Additionally in this review, we noted a lack of standardized language in defining missed doses. As our knowledge increases as to the most detrimental patterns of missed doses,^18^ knowing their causes will be critical to building interventions to better support patients who miss doses of therapy due to ADRs.^7^

We present detailed data as to whether specific ADRs were associated with missed doses in the underlying studies. Most ADRs (69%) reported belonged to 5 COS; we must consider whether this was due to selection bias (i.e. researchers monitoring for expected ADRs) or a true reflection of the ADRs presented. Regardless, the causation between specific ADRs and missed doses is multifaceted and should be examined holistically in relation to patients' health. For example, as people living with HIV are 16 times more likely to develop TB disease than those without HIV, it is important to consider the impact of comorbidities and the accompanying polypharmacy when examining ADRs.^2^

The included studies facilitate speculation that ADRs are more likely to cause patient‐originated missed doses (56%) than encourage HCPs to advise missed doses (33%). Perhaps HCPs have a greater understanding of the severity of ADRs or the possible consequences of treatment change compared to patients, thus are more reluctant to change treatment. Alternatively, perhaps HCPs are more likely to change or suspend single components of a regimen rather than the whole regimen, something not within the scope of this review. Additionally, the cause of the ADR, its severity (as well as its variability in severity), its reversibility or lack thereof, and whether treatments are available to alleviate it needs to be considered^8^; the heterogeneity of ADR presentation may explain the mixed evidence documented for whether a specific ADR was or was not associated with missed doses.

Moreover, some ADRs may be more easily recognized by patients (headache) and some by HCPs (asymptomatic elevated liver function tests). However, our study shows that some patient‐originated missed doses are associated with ADRs that are usually asymptomatic and only detected by a clinical test (e.g. hepatitis^19^, ^20^). This poses the question as to whether the patients being informed that they have an ADR can cause them to miss doses even in the absence of symptoms. The exact relationship between specific ADRs and missed doses is thus complicated and may partly be driven by anti‐TB medication being the perceived, as opposed to the actual, causative agent.^21^

To apply our findings to clinical practice whilst addressing the nuance of patient‐originated *vs*. HCP‐originated missed doses, investment in patient support is necessary. Two^22^, ^23^ of the 3 studies associating ADRs with a reduction in missed doses speculated that patients' increased engagement with HCPs due to ADRs led to fewer missed doses. This finding is consistent with other adherence‐promoting strategies.^4^ Understanding why certain ADRs cause doses to be missed where other ADRs do not is key to building effective strategies to minimize missed doses. Thus, patient support alongside shared decision‐making should be championed, with approaches tailored to local resources, HCP expertise and patients' circumstances.^24^, ^25^

A further dimension of nuance regarding patient‐ *vs*. HCP‐originated missed doses occurs when accounting for patients' DST profiles. Fewer studies reported ADRs associated with missed doses in DR populations compared with DS populations despite second‐line therapies having greater toxicity profiles than first‐line therapies. For example, the 2 *endocrine disorders* (hyperglycaemia and hypothyroidism) identified did not show evidence of increasing HCP‐originated missed doses. Since both these ADRs were identified in DR populations, alternative treatment options are limited and the risks associated with missing doses are elevated.^26^ Similar findings were found for ADRs within the COS of *ear and labyrinth disorders*. For such patients, it is possible that the route of administration (e.g., injectable *vs*. oral), which can vary depending on patients' DST profile, may affect the relationship between ADRs and missed doses, as well as patient‐originated *vs*. HCP‐originated missed doses.

Building upon initial findings outlined in this review, future research should focus on how the frequency and level of patient contact with HCP influences the relationship between missed doses and ADRs. Moreover, it would be useful to seek patient perspectives on the possible patterns of missed doses following different types of ADRs. For example, the patterns of patient‐originated missed doses may change based on how severe the patient deems the presenting ADR; maybe ADRs perceived as more serious by the patient will result in longer periods of patient‐originated missed doses compared with ADRs perceived as less serious.

As new DR‐TB therapies are introduced, patients on second‐line therapy will receive more effective treatments with reduced toxicity profiles.^27^ Stakeholders must monitor how new therapies with reduced toxicity profiles affect missed doses. Complacency as a result of this reduced profile regarding the attitude towards ADRs and missed doses must be avoided.

In this review, we explored the association between ADRs and missed doses of anti‐TB therapy. A range of study designs (both qualitative and quantitative) and locations provides wide insights into this relationship. We may have missed studies examining solely HCP‐originated missed doses where terminology around *treatment cessation* were used. Moreover, there was a large range in study size; data from larger studies can be interpreted with more certainty than those of a smaller size and may provide more generalizable data.

We were limited by the included studies, which presented few numerical analyses of the ADR–missed dose relationship. The severity of ADRs was unreported in most studies. The few numerical analyses also limited the conclusions we could draw; the data required us to analyse ADRs at a study level rather than a patient level. Moreover, since it was not possible to ascertain how the relative contribution of ADRs in causing missed doses was calculated in all relevant studies, we were required to accept the authors' finding, which may have been subjective or prone to bias. Discussions regarding ADRs and missed doses remain complex, with varying definitions and interpretations.

Despite these limitations, our scoping review is the first structured analysis of ADR‐missed dose relationships. It demonstrates extensive global evidence for the importance of ADRs as a primary driver of missed doses, and we present evidence to describe areas of variability within ADR–missed dose relationships (e.g. DST profile, originator of missed doses). We identify a critical knowledge gap: the absence of information regarding the patterns in which doses are missed due to ADRs. A much deeper understanding of how specific ADRs are related to specific patterns of missed doses is needed in order to generate effective interventions. Combined with upcoming less toxic drug regimens, and clinical and personal support from HCPs, these interventions will facilitate the development of targeted treatment support to improve treatment outcomes. Thus, personalized yet pragmatic patient support can be effectively and efficiently delivered.

## AUTHOR CONTRIBUTIONS

Eleanor G. Dixon, Derek J. Sloan, James W. Dear and Helen R. Stagg all made substantial contributions to the conception and design of the research. Brian Otaalo, Ashmika Motee, Shaista Rasool and Eleanor G. Dixon all substantially contributed to the acquisition of the data. Eleanor G. Dixon, Derek Sloan, James W. Dear and Helen R. Stagg interpreted the data. All authors read and approved the final manuscript.

## CONFLICT OF INTEREST STATEMENT

For the purpose of open access, the author has applied a creative commons attribution (CC BY) licence to any author accepted manuscript version arising from this submission.

H.R.S. declares funding through the UK Medical Research Council (MRC) in support of the present work and funding through the National Institute for Health Research (NIHR) Health Technology Assessment Programme, UK outside of the present work. H.R.S. declares other funding from the Latvian Society Against Tuberculosis (funding through Otsuka and Johnson and Johnson) outside the present work. All other authors declare no conflicts of interest.

## Supporting information


**TABLE S1.** Eligibility criteria.
**TABLE S2.** Search strategy—Medline.
**TABLE S3.** Search strategy—Embase.
**TABLE S4.** Search strategy—Web of Science.
**TABLE S5.** Standardised adverse drug reaction language.
**TABLE S6.** Overview of eligible papers.
**TABLE S7.** Overview of missed doses and ADR data.
**TABLE S8.** Specific adverse drug reactions reported in the context of missed doses.

## Data Availability

All data identified in this study are publicly available.
