# Supplementary material for: No action is without its side effects: Adverse drug reactions and missed doses of antituberculosis therapy, a scoping review
Source: Br J Clin Pharmacol. 2023 Oct 9;90(1):313–20. doi: 10.1111/bcp.15908 (PMC10952801; doi:10.1111/bcp.15908)
Supplement: Supplementary file 1 — TABLE S1. Eligibility criteria. TABLE S2. Search strategy—Medline. TABLE S3. Search strategy—Embase. TABLE S4. Search strategy—Web of Science. TABLE S5. Standardised adverse drug reaction language. TABLE S6. Overview of eligible papers. TABLE S7. Overview of missed doses and ADR data. TABLE S8. Specific adverse drug reactions reported in the context of missed doses. [file BCP-90-313-s001.docx]

**Table S1: Eligibility criteria**

|  | **Included** | **Excluded** |
| --- | --- | --- |
| **Population** | Human  Active TB disease  Drug-sensitive and drug-resistant disease  Primary data studies (including papers taken from review articles)  Any language  Published at any date | Latent TB infection  *M. bovis* infection  Single-patient case reports |
| **Exposure** | ADRs | ADRs not discussed |
| **Outcome** | Missed doses of all drugs in the regimen | Regimen-modified or single drug changes described |

The inclusion and exclusion criteria used in the study. ADR (adverse drug reaction); TB (tuberculosis).

**Table S2: Search Strategy- Medline**

| **Search Line** | **Search Term** | **Number of Hits** |
| --- | --- | --- |
| 1 | Tuberculosis/ or Antitubercular Agents/ | 136294 |
| 2 | TB.mp. [mp=title, abstract, original title, name of substance word, subject heading word, floating sub-heading word, keyword heading word, organism supplementary concept word, protocol supplementary concept word, rare disease supplementary concept word, unique identifier, synonyms] | 50952 |
| 3 | 1 or 2 | 163132 |
| 4 | Medication Adherence/ or "Treatment Adherence and Compliance"/ or Treatment Refusal/ | 34607 |
| 5 | (adhere* or complian* or non-adhere* or nonadhere* or concordan* or non-concordan*).mp. [mp=title, abstract, original title, name of substance word, subject heading word, floating sub-heading word, keyword heading word, organism supplementary concept word, protocol supplementary concept word, rare disease supplementary concept word, unique identifier, synonyms] | 417541 |
| 6 | "Lost to Follow-Up"/ | 1411 |
| 7 | (LTFU or "los* to follow-up" or "los* to follow up" or LFU or default*).mp. [mp=title, abstract, original title, name of substance word, subject heading word, floating sub-heading word, keyword heading word, organism supplementary concept word, protocol supplementary concept word, rare disease supplementary concept word, unique identifier, synonyms] | 38416 |
| 8 | (perception* or perspective* or "treatment challenge*" or "treatment behavio*" or "treatment-related factor*" or "treatment related factor*").mp. [mp=title, abstract, original title, name of substance word, subject heading word, floating sub-heading word, keyword heading word, organism supplementary concept word, protocol supplementary concept word, rare disease supplementary concept word, unique identifier, synonyms] | 705618 |
| 9 | 4 or 5 or 6 or 7 or 8 | 1148040 |
| 10 | 3 and 9 | 7579 |
| 11 | limit 10 to humans | 7261 |

Medline was searched on 3rd November 2021 using Ovid MEDLINE(R) <1946 to October Week 4 2021>

**Table S3: Search Strategy- Embase**

| **Search Line** | **Search Term** | **Number of Hits** |
| --- | --- | --- |
| 1 | tuberculosis/ | 116522 |
| 2 | tuberculostatic agent/ | 34378 |
| 3 | TB.mp. [mp=title, abstract, heading word, drug trade name, original title, device manufacturer, drug manufacturer, device trade name, keyword heading word, floating subheading word, candidate term word] | 79412 |
| 4 | 1 or 2 or 3 | 182276 |
| 5 | patient compliance/ | 140246 |
| 6 | medication compliance/ | 37822 |
| 7 | treatment refusal/ | 20460 |
| 8 | (adhere* or complian* or non-adhere* or nonadhere* or concordan* or non-concordan*).mp. [mp=title, abstract, heading word, drug trade name, original title, device manufacturer, drug manufacturer, device trade name, keyword heading word, floating subheading word, candidate term word] | 739490 |
| 9 | (LTFU or "los* to follow-up" or "los* to follow up" or LFU or default*).mp. [mp=title, abstract, heading word, drug trade name, original title, device manufacturer, drug manufacturer, device trade name, keyword heading word, floating subheading word, candidate term word] | 76597 |
| 10 | (perception* or perspective* or "treatment challenge*" or "treatment behavio*" or "treatment-related factor*" or "treatment related factor*").mp. [mp=title, abstract, heading word, drug trade name, original title, device manufacturer, drug manufacturer, device trade name, keyword heading word, floating subheading word, candidate term word] | 895865 |
| 11 | 5 or 6 or 7 or 8 or 9 or 10 | 1686341 |
| 12 | 4 and 11 | 14044 |
| 13 | limit 12 to human | 12808 |

Embase was searched on 3rd November 2021 using Ovid Embase <1974 to 2021 November 01>

**Table S4 Search Strategy- Web of Science**

| **Search Line** | **Search Term** | **Number of hits** |
| --- | --- | --- |
| 1 | TS=(TB or Tuberculosis) | 228597 |
| 2 | TS=(adhere* or complian* or non-adhere* or nonadhere* or concordan* or non-condordan*) | 586460 |
| 3 | TS=(LTFU or ‘los* to follow-up” or “los* to follow up” or LFU or default*) | 76860 |
| 4 | TS=(perception* or perspective* or “treatment challenge*” or “treatment behavio*” or “treatment-related factor*” or “treatment related factor*”) | 1767477 |
| 5 | #2 or #3 or #4 | 2391063 |
| 6 | #1 and #5 | 10290 |

Web of Science was searched on 3rd November 2021.

## Text 1: Search strategy

Search terms were decided after several rounds of exploratory searches, during which we used the identification of relevant studies as a mechanism to assess the accuracy of the search. As documented in the introduction, non-adherence to treatment is a known form of missed doses, so we included terms around this (adherence, compliance, concordance). Additionally, as the standard tuberculosis (TB) surveillance outcome ‘loss to follow-up’ (LTFU) documents a form of missed doses through early treatment cessation, terms around this concept were also included (1). Finally, to capture both qualitative papers and those that focussed on healthcare professional (HCP)-initiated missed doses, we included terms we found to be used in such studies. Terms around adverse drug reactions (ADRs) were not included because exploratory searches indicated that broader search terms relating to treatment challenges generated more relevant hits than ADR-specific language.

No limit was placed on which fields the search terms could appear. In line with the inclusion criteria, the final search results were limited to papers with human data only.

## Text 2: Classifying disease drug sensitivity

Patients described as drug naïve were classified as having drug sensitive (DS) disease. In cases where the drug susceptibility testing (DST) profile was not stated, drug regimen was used as a proxy and *vice versa*. Patients prescribed the first-line regimen (2HRZE/4HR (two months of isoniazid, rifampicin, pyrazinamide and ethambutol followed by four months of isoniazid and rifampicin)) (2, 3) or variations of this regimen were assumed to have DS TB. Patients prescribed regimens which were clearly identified with any form of drug resistance (4, 5), were assumed to have drug resistant (DR) TB. Studies with multiple regimens that did not clearly indicate a single DST profile, or where DST profile and regimen choice were unknown were assumed to have been conducted in populations with mixed DST profiles.

## Text 3: Relative burden

A study was included as describing ADRs as a primary reason for missed doses in the following circumstances: the study stated that ADRs were the main, major, or primary reason for missed doses or the study listed many reasons with rankings/ percentages as to the reasons for missed doses and ADRs were either the first or second reason in terms of prominence.

**Table S5: Standardised adverse drug reaction language**

| **COS** | **MedDRA term (n=68)** | **Language used in the studies (n=136)** |
| --- | --- | --- |
| **Blood and Lymphatic System Disorders** | Anaemia | Anaemia  Moderate anaemia |
|  | Eosinophilia | Eosinophilia |
|  | Haematological toxicity | Haematological toxicity |
|  | Leukopenia | Leukopenia |
|  | Thrombocytopenia | Thrombocytopenia |
| **Cardiac Disorders** | Bradycardia | Bradycardia |
| **Ear and Labyrinth Disorders** | Hearing losses | Hearing loss  Mild hearing loss |
|  | Ototoxicity | Ototoxicity |
| **Endocrine Disorders** | Hyperglycaemia | Hyperglycaemia |
|  | Hypothyroidism | Hypothyroidism |
| **Eye Disorders** | Eye disorders | Eyes |
|  | Optic neuritis | Optic neuritis |
|  | Visual acuity reduced | Decreased visual acuity |
| **Gastrointestinal Disorders** | Abdominal discomfort | Gastrointestinal bleedings  Gastrointestinal discomfort  Gastrointestinal disturbances  Gastrointestinal reaction  Gastrointestinal toxicity  Gastrointestinal upset  Stomach problems |
|  | Abdominal pain | Abdominal pain  Abdominal pain/ cramps  Feel pain in my stomach  Stomach ache |
|  | Constipation | Constipated |
|  | Diarrhoea | Diarrhoea |
|  | Gastritis | Gastritis  Mild gastritis  Severe gastritis |
|  | Gastrointestinal nonspecific dysfunction | Digestive disorders  Severe digestive disorders |
|  | Nausea and vomiting symptoms | Gastritis (nausea, vomiting)  Nausea  Nausea/ vomiting  Sick and nauseous  Vomit  Vomiting |
|  | Taste disorder | No food to be of good taste |
| **General Disorders and Administration Site Conditions** | Asthenia | Feeling weak  General weakness  Physical indisposition  Weakness  Weakness/ lethargy |
|  | Pain | Body pain  Chest pain  Pain  Whole body would ache |
| **Hepatobiliary Disorders** | Acute hepatic failure | Acute liver failure |
|  | Elevated liver function test | Elevated LFTs  Elevated liver function test  High transaminase levels  Mild transient elevation in serum AST or ALT  Mildly elevated total bilirubin  Transaminase elevation |
|  | Hepatic cholestatic | Cholestatic hepatitis |
|  | Hepatic cytolysis | Moderate hepatic cytolysis |
|  | Hepatic disorders | Liver |
|  | Hepatitis | Drug induced hepatitis  Fulminant hepatitis drug induced  Hepatitis |
|  | Hepatotoxicity | Hepatotoxicity  Liver toxicity  Mild hepatotoxicity  Sever hepatotoxicity |
|  | Jaundice | Jaundice |
|  | Liver injury | Liver damage |
| **Immune System Disorders** | Dermatitis allergic | Allergic rash |
|  | Hypersensitivity reaction | Allergic reactions  Hypersensitivity reaction |
| **Metabolism and Nutrition Disorders** | Decreased appetite | Lack of appetite  Loss of appetite |
|  | Feeding disorder | Hunger (unable to eat) |
|  | Hyperuricemia | Hyperuricemia |
| **Musculoskeletal and Connective Tissue Disorders** | Joint disorders | Arthralgia  Arthralgia/ arthritis  Joint pain  Osteoarthritic |
|  | Myalgia | Myalgia |
|  | Myopathy | Muscle weakness |
| **Nervous System Disorders** | Dizziness | Dizziness  Dizzy  Giddiness  Lightheadedness |
|  | Encephalopathies toxic and metabolic | Hepatic encepholaphathy |
|  | Headache | Central nervous system (seizure of intractable headache)  Head reeling  Headache  Headaches  Severe headache |
|  | Nervous system disorders | Neuro  Neurological toxicity  Peripheral nervous system |
|  | Paraesthesia | Paraesthesia in lower limbs |
|  | Peripheral neuropathy | Neuropathy  Peripheral neuropathy |
|  | Syncope | Syncope |
|  | Vertigo | Vertigo |
| **Psychiatric Disorders** | Anxiety | Anxiety |
|  | Confusion and disorientation | Confusion  Intense confusion |
|  | Depression | Depression |
|  | Disturbances in thinking and perception | Disturbed mind |
|  | Insomnia | Couldn't sleep at night  Insomnia |
|  | Psychiatric disorders | Aggravation of underlying psychiatric disorder  Psychotic systems  Psychiatric  Psychiatric conditions |
|  | Restlessness | Restlessness |
|  | Sleep disorders | Night disturbances |
|  | Sleep terror | Nightmares |
|  | Suicidal ideation | Suicidal thoughts |
| **Renal and Urinary Disorders** | Chromaturia | Discolouration of urine |
|  | Nephropathy toxic | Nephrotoxicity  Renal toxicity |
|  | Renal disorders | Kidney  Mild renal dysfunction |
|  | Renal impairment | Renal impairment |
| **Skin and Subcutaneous Tissue Disorders** | Dermatotoxicity | Dermatological effects  Dermatotoxicity  Dermatological toxicity  Skin  Toxiderma rifampicin |
|  | Flushing | Flush syndrome |
|  | Pallor | Pallor |
|  | Pigmentation disorder | Skin pigmentation |
|  | Pruritus | Generalised pruritus  Itching  Itching sensation/ skin eruption  Itching/ skin rash  Transient generalised pruritus |
|  | Rash | Rashes  Skin rash  Transient skin rash |

Adverse drug reaction (ADR) terms used in the studies was standardised using Medical Dictionary for Regulatory Activities terminology (MedDRA). ADR terms were grouped based on their class organ system (COS). Some ADRs were reported on a more general level (e.g. ‘haematological disorders’) and therefore the term reported could include many, more specific ADRs (e.g. ‘anaemia’). MedDRA terms were allocated by two authors independently before confirming the term. ADR (adverse drug reaction); COS (class organ system); MedDRA (Medical Dictionary for Regulatory Activities); n (number).

**Table S6: Overview of eligible papers**

| **Study ID** | **About the study** | | | **Study population** | | | | **TB information** | | |
| --- | --- | --- | --- | --- | --- | --- | --- | --- | --- | --- |
|  | **Study design** | **Date of data collection** | **Country of study** | **Number of participants** | **Age (years)** | **% Female** | **Comorbidities** | **Site of disease** | **Disease sensitivity** | **Drug regimen** |
| Agudelo 2021 (6) | Prospective cohort | 2014- 2015 | Colombia | 128 | 38 (mean) | 20 | Anaemia, HIV, depressive disorder, viral hepatitis, chronic lung disease, epilepsy, chronic kidney disease, diabetes mellitus | P, E, P&E | - | Various regimens |
| Akeju 2017 (7) | Qualitative | - | South Africa | 22 | ≥18 | - | - | - | DN, DS | - |
| Al-Dossary 2002 (8) | Prospective cohort | 1994- 2000 | USA | 185 | 3 (median, paediatric) | 51 | None have HIV | P, E | DS | Variation of 2HRZE/4HR |
| Ali 2017 (9) | Case-control | 2011- 2011 | Sudan | Defaulters: 105 Control: 210 | >15 | Defaulters: 30 Control: 40 | Chronic disease | P, E | DS | Variation of 2HRZE/4HR |
| Amalba 2021 (10) | Cross-sectional | 2017- 2018 | Ghana | 66 | 8 - 95 | 50 | None | - | DN | 2HRZE/4HR |
| Amuha 2009 (11) | Cross-sectional (qualitative and quantitative) | 2008 | Uganda | 140 | 32 (median) | 43 | All have HIV | - | - | - |
| Auchynka 2021 (12) | Retrospective cohort | 2016- 2018 | Belarus | 125 | 43 (mean) | 28 | HIV, diabetes, hypertension, HBV, HCV, baseline cardiovascular disorders, ECG abnormalities | P, P & E | MDR, XDR | Variation of WHO recommended MDR-TB regimen |
| Barrah 2017 (13) | Retrospective cohort | - | Tunisia | 41 | - | - | All on dialysis | - | - | 2HRZE/4HR |
| Bartacek 2009 (14) | RCT | 2003- 2004 | 5 countries | 1159 | ≥15 | 32 | None | P | DN | 2HRZE/4HR |
| Bastard 2015 (15) | Retrospective cohort | 2002- 2010 | Armenia and Abkhazia | 393 | 38 (mean) | 17 | Alcohol, diabetes | - | MDR | Various regimens |
| Burton 2011 (16) | Retrospective cohort | 2009 | Ghana | 599 | 42 (mean) | 38 | HIV | - | - | - |
| Caceres 2007 (17) | Prospective cohort | 2002- 2003 | Colombia | 261 | 38 (median) | 38 | - | - | - | - |
| Campani 2011 (18) | Case-control | 2004-2006 | Brazil | Non-compliant: 218 Control: 218 | Non-compliant: 33 (mean) Controls: 38 (mean) | Non-compliant: 31 Controls: 36 | HIV | P | - | Variation of 2HRZE/4HR |
| Canete 1994 (19) | Prospective cohort | 1985- 1992 | Spain | 130 | 27 (mean) | 47 | None HIV | E | - | Variation of 2HRZE/4HR |
| Cardoso 2017 (20) | Retrospective cohort | 2012- 2014 | Brazil | 279 | 40 (mean) | 44 | HIV, alcohol, tobacco | P, E | - | 2HRZE/4HR |
| Cator 2002 (21) | Retrospective cohort | 1992- 1995 | Canada | 385 | 0-50 | 44 | HIV | - | - | - |
| Chang 2004 (22) | Case control | 1999 | Hong Kong | Defaulters: 102 Controls: 306 | 49 (mean) | 14 | Chronic disease history, hepatitis | - | - | - |
| Chee 2006 (23) | Retrospective cohort | 2001- 2002 | Singapore | 1538 | - | - | - | - | - | - |
| Cherkaoui 2014 (24) | Case-control | 2010- 2011 | Morocco | Defaulters: 91 Controls: 186 | Adult | 31 | Illicit drug use, mental illness, chronic illness. None have HIV. | P, E | - | - |
| Chida 2015 (25) | Qualitative | 2007- 2014 | Pakistan | Quantitative: 2120 Qualitative: 21 | ≥18 | Qualitative: 53 | - | - | DS | 2HRZE/4HR; variation of 2HRZE/4HR |
| Chino 2016 (26) | Retrospective cohort | 2011- 2014 | Japan | 298 | 84 (median) | 34 | Hypertension, diabetes, malignancy, cerebrovascular disease | P | DS, DR | Various regimens; variation of 2HRZE/4HR |
| Ciza 2020 (27) | Retrospective cohort | 2013- 2017 | Burundi | 225 | 21-80 | 39 | HIV | P | DR | Variation of 2HRZE/4HR |
| Colmenero 1997 (28) | Prospective cohort | 1993- 1994 | Spain | 84 | 30 (mean) | 38 | HIV | P, E | DN, DS | Variation of 2HRZE/4HR |
| Combs 1990 (29) | RCT | 1981- unknown | USA | 1451 | ≥18 | 22 | - | P | DS, DR | Variation of 2HRZE/4HR |
| Dalay 2017 (30) | Retrospective cohort | 2015 | Philippines | 160 | 90% were >15 | - | Diabetes, HIV | - | MDR | - |
| Das Shukla 2019 (31) | Cross-sectional | 2015- 2016 | India | 116 | >18 | - | None have HIV or are pregnant | P | MDR, non-MDR | WHO recommended MDR-TB regimen |
| Davtyan 2019 (32) | Cross-sectional | 2014- 2015 | Armenia | 505 | 46 (mean) | 24 | - | P, E | DS, DR | - |
| Deshmukh 2015 (33) | Qualitative | 2012- 2013 | India | 20 | 23-53 | 25 | - | - | MDR | - |
| Dey 2021 (34) | Retrospective cohort | 2018- 2019 | India | 7505 | 40 (median) | 31 | Alcohol, diabetes, HIV, tobacco | - | DS, DR | - |
| Dhingra 2004 (35) | Prospective cohort | 2002- 2003 | India | 1195 | - | - | - | - | - | Variation of 2HRZE/4HR |
| Donald 1998 (36) | Prospective cohort | 1991- 1994 | South Africa | 95 | 17- 38 months (medians, paediatric) | - | - | Me | - | Variation of 2HRZE/4HR |
| Drobac 2006 (37) | Retrospective cohort | 1999- 2003 | Peru | 38 | 11 (median, paediatric) | 55 | Anaemia, malnutrition, HIV, other | P, E, M | MDR | Various regimens |
| Duraisamy 2014 (38) | Retrospective cohort | 2009- 2010 | India | 179 | 45 (median) | 22 | HIV | - | MDR | WHO recommended MDR-TB regimen |
| Elbireer 2011 (39) | Case-control | 2009 | Uganda | 344 | 36 (mean) | 49 | All have HIV | - | - | - |
| Fang 2019 (40) | Cross-sectional | 2014 | China | 262 | 51 (mean) | 25 | - | P | - | - |
| Ferrer 1991 (41) | Prospective cohort | 1987- 1988 | Chile | 176 | >15 | 51 | - | - | - | - |
| Franck 2014 (42) | Qualitative | 2009- 2010 | South Africa | 20 | 7 - 17 (paediatric) | 70 | HIV | - | MDR | - |
| Fry 2005 (43) | Cross-sectional | 2003 | Russia | 100 | Group 1: 30 (mean) Group 2: 41 (mean) | Group 1: 17  Group 2: 15 | - | - | - | - |
| Furin 2001 (44) | Retrospective cohort | 1996- 1998 | Peru | 60 | 26 (median) | 48 | Depression, HIV, diabetes, epilepsy, aortic stenosis, prostatic hypertrophy, cerebrovascular disease | - | MDR | Various regimens |
| Gafar 2019 (45) | Retrospective cohort | 1993- 2018 | Netherlands | 3396 | 0-18 | 44 | - | P, E | DS, DR | - |
| Galstyan 2018 (46) | Prospective cohort | 2015- 2017 | - | 28 | 24- 57 | 32 | HIV | D, P | XDR, MDR | Various regimens |
| Garazzino 2014 (47) | Prospective cohort | 2007- 2012 | Italy | 9 | 6 months- 13 | 66 | - | P | DS, DR | Various regimens |
| Gebremariam 2010 (48) | Qualitative | 2008 | Ethiopia | 15 | - | - | HIV | - | - | - |
| Gonzalez 2012 (49) | Retrospective cohort | 1979- 2009 | Argentina | 1065 | >15 | 37 | None have HIV | P, E | - | Variation of 2HRZE/4HR |
| Gorityala 2015 (50) | Cross-sectional | - | India | 107 | ≥18 | 8 | - | P | - | - |
| Gualano 2019 (51) | Retrospective cohort | 2008- 2016 | Italy | 74 | 32 (mean) | 42 | Chronic HBV or HCV, diabetes, HIV, others | P, E, P&E | MDR | Various regimens |
| Gugssa Boru 2017 (52) | Qualitative | 2014 | Ethiopia | 22 | 36 (mean) | 64 | - | - | - | - |
| Gunawan 2014 (53) | Case-control | 2013- 2014 | Indonesia | Default: 63 Controls: 63 | - | Default: 36 Controls: 63 | - | P | - | - |
| Gupta 1992 (54) | RCT | - | India | 60 | 15 - 55 | - | - | P | DN | Variation of 2HRZE/4HR |
| Hamadah 2016 (55) | Retrospective cohort | 2002- 2014 | USA | 5 | 60 (median) | 40 | All haemodialysis patients | - | - | 2HRZE/4HR |
| Helegbe 2019 (56) | Cross-sectional | 2014 | Ghana | 95 | - | - | - | - | - | - |
| Hirpa 2013 (57) | Case-control | 2011- 2012 | Ethiopia | Cases: 134 Control: 134 | Cases: 25 (mean) Control: 31 (mean) | Cases: 40 Controls: 53 | HIV, other | - | DS (controls) MDR (cases) | - |
| Hoshino 2007 (58) | Retrospective cohort | 2004 | Japan | 194 | - | - | - | P | DS, MDR | - |
| Hu 2005 (59) | Cross-sectional | 2001 | Taiwan | Group 1: 450 Group 2: 400 | - | Group 1: 32 Group 2: 26 | - | P | - | - |
| Isaakidis 2013 (60) | Retrospective cohort | 2007- 2013 | India | 11 | 16 (median) | 54 | All have HIV | P, P & E | MDR | WHO recommended MDR-TB regimen |
| Jaber 2018 (61) | Prospective cohort | 2013- 2015 | Yemen | 413 | ≥16 | 35 | ‘Comorbidities' | P, E | DS | 2HRZE/4HR |
| Jaggarajamma 2007 (62) | Retrospective cohort | 1999- 2001 | India | 938 | - | 25 | - | P, E | - | - |
| Jaiswal 2021 (63) | Cross-sectional | 2020 | India | 55 | 15-75 | 20 | No COVID or psychosis | x | DN | 2HRZE/4HR |
| Janakan 2008 (64) | Prospective cohort | 2002 | Sri Lanka | 326 | >15 | 28 | - | P | DN | - |
| Jittimanee 2007 (65) | Prospective cohort | 2003- 2005 | Thailand | 160 | 39 (mean) | 44 | HIV | - | - | - |
| Kan 1985 (66) | Prospective cohort | - | China | 229 | 15- 70 | 49 | No renal or hepatic disease | P | DN (DS, DR) | Various regimens |
| Kandel 2008 (67) | Cross-sectional | 2001- 2003 | South Africa | 255 | - | - | - | - | - | - |
| Keshavjee 2012 (68) | Retrospective Cohort | 2000- 2004 | Russia | 568 | 34 (median) | 18 | Respiratory insufficiency, gastritis/ ulcer, cerebrovascular disease, psychiatric disorder, diabetes mellitus, seizure disorder, renal insufficiency, HIV | - | MDR | Various regimens |
| Khan 2015 (69) | Cross-sectional | 2013- 2014 | India | 12 | ≥15 | 25 | - | - | - | Variation of 2HRZE/4HR |
| Kim 2020 (70) | Cross-sectional | 2015- 2017 | South Korea | 780 | - | ~40 | - | P, P & E | DS | - |
| Kiria 2018 (71) | Retrospective cohort | 2014- 2017 | Georgia | 18 | 43 (mean) | 0 | HCV. All have HIV | P | PXTB, XTB | Variation of WHO recommended MDR-TB regimen |
| Kisambu 2014 (72) | Cross-sectional | 2008- 2009 | Uganda | 126 | ≥18 | 54 | - | - | - | - |
| Lienhardt 2011 (73) | RCT | 2003- 2008 | 9 countries | 1170 | ≥18 | 33 | None | P | DN, DS | 2HRZE/4HR |
| Medical Research Council 1989 (74) | Retrospective cohort | 1983- 1983 | UK | 390 | ≤14 | - | - | P, E, P&E | - | Variation of 2HRZE/4HR |
| Muture 2011 (75) | Case-control | 2006- 2008 | Kenya | 1978 | Defaulter: 31 (mean) Controls: 30 (mean) | Defaulters: 41 Controls: 47 | HIV | P, E | - | - |
| Nandadeva 2014 (76) | Cross-sectional | 2013 | Sri Lanka | 30 | 48 (mean) | 37 | Hypertension, chronic renal failure, diabetes, ischemic heart disease | P, E | - | - |
| Orofino 2012 (77) | Retrospective cohort | 2004- 2006 | Brazil | 311 | 39 (mean) | - | HIV | - | - | Variation of 2HRZE/4HR |
| Park 2015 (78) | Case-control | - | South Korea | 150 | - | - | - | - | - | Variation of 2HRZE/4HR |
| Patel 2020 (79) | Prospective cohort | 2019 | India | 100 | - | 16 | None are 'critically ill' | - | - | - |
| Piubello 2014 (80) | Prospective cohort | 2008- 2010 | Niger | 65 | 31 (median) | 19 | HIV. None are pregnant or have severe liver insufficiency. | - | MDR | Various regimens |
| Ranawaka 2021 (81) | Cross-sectional | 2018 | Sri Lanka | 252 | ≥18 | 37 | Alcohol, smoking | - | - | 2HRZE/4HR |
| Ranganath 2021 (82) | Case-control | 2019 | India | Non-adherent: 125  Controls: 375 | 40 (mean) | Non-adherent: 34 Controls 37 | All have HIV | P, E | - | - |
| Reves 2011 (83) | Prospective cohort | 1999- 2004 | USA | 98 | - | - | - | P | MDR, DR | Variation of 2HRZE/4HR |
| Reyes-Guillen 2008 (84) | Qualitative | 2002- 2003 | Mexico | 9 | 22-80 | 11 | - | P | - | - |
| Rodrigues 2010 (85) | Prospective cohort (qualitative) | - | Brazil | 45 | - | - | All have HIV | - | - | - |
| Rupani 2020 (86) | Cross-sectional | 2016 | India | 94 | >10 | 32 | Diabetes, HIV, chronic renal failure | - | MDR | - |
| Saleem 2015 (87) | Cross-sectional | 2015 | Pakistan | 80 | 15- 60 | 36 | - | - | - | - |
| Sanchez-Padilla 2014 (88) | Retrospective cohort with qualitative | 2005- 2011 | Armenia | 381 | No defaulter: 37 (median) Defaulter: 40 (median) | No defaulter: 24 Defaulter: 8 | Illicit drug use, alcohol | - | DR, MDR | - |
| Santos 2018 (89) | Retrospective cohort | 2012- 2017 | Brazil | 50 | 40 (mean) | 38 | Illicit drug use, alcoholism, AIDS, smoking, cancer, diabetes, viral hepatitis, other | - | DR, MDR, XDR | - |
| Sarpal 2014 (90) | Prospective cohort | 2010- 2012 | India | 545 | ≥5 | 3 | - | P, E | - | Variation of WHO recommended MDR-TB regimen |
| Satti 2012 (91) | Retrospective cohort | 2007- 2011 | Lesotho | 19 | 8 (median, paediatric) | - | HIV, malnutrition | P, E, M | MDR | Various regimens |
| Setiawan 2019 (92) | Retrospective cohort | 2014 | Indonesia | 172 | ≥15 | 39 | General comorbidities | P | - | Variation of 2HRZE/4HR |
| Shringarpure 2016 (93) | Qualitative | 2013- 2014 | India | 32 | 13-55 | 28 | - | - | DR, MDR | - |
| Singh 2017 (94) | Prospective cohort | 2014- 2015 | India | 100 | 15-80 | - | None | - | DS | 2HRZE/4HR |
| Skrahina 2020 (95) | Retrospective cohort | 2018- 2020 | Belarus | 300 | 45 (median) | 21 | Alcoholism, HCV, HIV, diabetes | - | MDR | Variation of WHO recommended MDR-TB regimen |
| Snene 2015 (96) | Retrospective cohort | 2009- 2013 | Tunisia | 54 | 38 (mean) | - | - | - | MDR | - |
| Su 2002 (97) | RCT | 1997- 1998 | Taiwan | 105 | ≥18 | 11 | - | P | DN | Variation of 2HRZE/4HR |
| Sun 2021 (98) | Prospective cohort | 2017- 2018 | China | 114 | 18- 65 | 47 | None | P | MDR | WHO recommended MDR-TB regimen |
| Taufiqi 2014 (99) | Retrospective cohort | 2011- 2013 | Indonesia | 19 | 42.1% were 51-60 | 37 | - | - | MDR | - |
| Tekle 2002 (100) | Case control | 1997- 2002 | Ethiopia | 1367 | 0- ≥60 | 45 | - | P, E | - | Variation of 2HRZE/4HR |
| Tesfahuneygn 2015 (101) | Cross- sectional | 2012- 2013 | Ethiopia | 200 | >15 years | 42 | HIV, alcohol | P, E | - | - |
| Tsakalidis 1992 (102) | Prospective cohort | 1988- 1991 | Greece | 36 | 6 (mean, paediatric) | 36 | - | P, E | - | Variation of 2HRZE/4HR |
| Ukoha-Kalu 2020 (103) | Cross-sectional | 2018 | Nigeria | 60 | ≥15 | 37 | All have HIV | - | - | - |
| Veeramani 2015 (104) | Prospective cohort | 2014 | India | 282 | - | 28 | - | - | - | - |
| Walker 2019 (105) | Multiple cohorts | - | 22 countries | 4099 | 0 - 99 | 31 | HIV | - | MDR | - |
| Wares 2003 (106) | Qualitative | 2000 | Nepal | 61 | ≥15 | 28 | - | P, E | - | - |
| Wobudeya 2019 (107) | Retrospective cohort | 2010- 2015 | Uganda | 516 | 36 months (median, paediatric) | 45 | HIV | P, E | DS | Variation of 2HRZE/4HR |
| Wohlleben 2017 (108) | Case-control | 2011-2012 | Tajikistan | LTFU: 300 Controls: 592 | - | 33- 47 | - | P, E | - | Variation of 2HRZE/4HR |
| Woldeyohannes 2021 (109) | Retrospective cohort | 2012- 2017 | Ethiopia | 406 | 28 (median) | 41 | HIV, chronic diseases, coinfections | P | DR | - |
| Xing 2021 (110) | Qualitative | 2017- 2018 | China | 132 | ≥15 | 36 | - | - | MDR | - |
| Xu 2009 (111) | Prospective cohort (qualitative and quantitative) | 2006- unknown | China | 670 | 51 | 27 | - | P | - | - |
| Zhang 1996 (112) | RCT | 1992- 1993 | China | 209 | ≥15 | 36 | None | P | DN | Variation of 2HRZE/4HR |
| Zhang 2020 (113) | Qualitative | 2019 | China | 17 | 30 (mean) | 59 | None | P | - | - |

A summary of the eligible papers in the scoping review. Number of participants was the number of participants at the start of the study. The range in sample size was large (9 to 7505 participants) which reflected the range in study designs, study populations and study locations. Comorbidities specifically documented in the underlying studies were extracted; ‘all’ indicates where they applied to all study participants and ‘none’ where there were not relevant to any individuals in the study population. In Ali 2017, Campani 2011, Chang 2004, Cherkaoui 2014, Gunawan 2014, Ranganath 2021 and Wohlleben 2017, ‘defaulters’, ‘non-compliant’, ‘non-adherent’ or ‘LTFU’ (lost to follow up) tuberculosis (TB) patients were the ‘cases’ and ‘controls’ were TB patients who were registered as being cured or having completed treatment. In Hirpa 2013, ‘cases’ were confirmed multi-drug resistant (MDR) TB patients who were undergoing treatment and ‘controls’ were patients who had been on ‘first-line anti-TB treatment’ and were registered as being cured or having completed treatment. In Hu 2005, ‘Group 1’ were patients ‘adherent’ to their anti-TB treatment and ‘Group 2’ were patients ‘non-adherent’ to their anti-TB treatment. In Rodrigues 2010, the participants were HCP reporting the experiences of 45 patients. – (data not presented in study);

2HRZE/4HR (two months of isoniazid, rifampicin, pyrazinamide and ethambutol followed by four months of isoniazid and rifampicin (3); AIDS (acquired immune deficiency syndrome); D (disseminated); DN (drug naïve); DR (drug-resistant); DS (drug-sensitive); ECG (electrocardiogram); E (extrapulmonary); HBV (hepatitis B virus); HCV (hepatitis C virus); HCP (healthcare professional); HIV (human immunodeficiency virus); LTFU (lost/ loss to follow up); M (miliary); MDR (multi-drug resistant); Me (meningitis); P (pulmonary); P&E (pulmonary and extrapulmonary; PXDR (pre-extensively drug-resistant); RCT (randomised control trial); variation of 2HRZE/4HR (therapy was based on 2HRZE/4HR but had small changes such as the removal of one of the drugs, a shorter/ longer treatment period, etc.; variation of WHO recommended MDR-TB regimen (a variation of anti-MDR-TB therapy as recommended by the WHO (4)); various regimens (therapy was either prescribed on an individual basis or there were many variations of treatment options across study participants; WHO (World Health Organization); WHO recommended MDR-TB regimen (anti-MDR-TB therapy as recommended by the WHO (fluoroquinolone and injectable drugs) (4); XDR (extensively drug-resistant).

**Table S7: Overview of missed-doses and ADR data**

| **Study ID** | **Definitions** | | **Sources of information** | | **Outcome** | **Exposure** | **Relationship** |
| --- | --- | --- | --- | --- | --- | --- | --- |
|  | **Summary of missed doses definition** | **Summary of ADR definition** | **Source of missed doses information** | **Method of measuring ADRs** | **Number of people who missed doses (n (%))** | **Number of people with ADRs (n (%))** | **Number of people who experienced ADRs and missing doses** |
| Agudelo 2021 (6) | Medication suspended ** | Liver function tests; toxicities that appeared after the start of anti-TB treatment and resolved after stopping it. | Study or clinical notes | Study or clinical notes | - | 15 (12) | 6 |
| Akeju 2017 (7) | Failure to comply with therapy as prescribed * | - | Self-report | Records in database, study notes or registry | - | - | - |
| Al-Dossary 2002 (8) | Treatment interruption ** | - | Study or clinical notes | Study or clinical notes | - | - | 2 |
| Ali 2017 (9) | WHO definition * | - | Study or clinical notes | Self-report and study or clinical notes | 105 (33) | 120 (38) | 55 |
| Amalba 2021 (10) | Missing doses and default *** | - | Self-report | Self-report | 34 (52) | 51 (77) | 26 |
| Amuha 2009 (11) | Discontinuation * | - | Study or clinical notes | Self-report and study or clinical notes | 35 (25) | - | - |
| Auchynka 2021 (12) | Discontinuation ** | Graded using CTCAE (common terminology criteria for adverse events) | Records in database, study notes or registry | Records in database, study notes or registry | - | - | - |
| Barrah 2017 (13) | Abstain and treatment stop *** | - | Records in database, study notes or registry | Records in database, study notes or registry | 14 (34) | 19 (46) | 7 |
| Bartacek 2009 (14) | Missed intake of medication for >3 consecutive days in intensive phase and >7 in continuation phase * | - | Study or clinical notes | Study or clinical notes | - | 246 (21) | 40 |
| Bastard 2015 (15) | Therapy stopped ≥2 consecutive days ** | - | Records in database, study notes or registry | Records in database, study notes or registry | - | - | - |
| Burton 2011 (16) | Default and discontinuation * | - | Records in database, study notes or registry | Records in database, study notes or registry | - | - | 6 |
| Caceres 2007 (17) | Treatment abandoned * | - | Records in database, study notes or registry | Records in database, study notes or registry | - | - | - |
| Campani 2011 (18) | Failing to report for more than 60 days after the last visit and thereby remaining off medication for at least 30 days * | - | Study or clinical notes | Study or clinical notes | 218 (50) | - | - |
| Canete 1994 (19) | Non-continuation of treatment * | - | Study or clinical notes | Study or clinical notes | - | 27 (21) | 3 |
| Cardoso 2017 (20) | WHO definition * | Treatment interrupted by ADRs | Records in database, study notes or registry | Records in database, study notes or registry | 40 (14) | - | - |
| Cator 2002 (21) | WHO definition * | - | Records in database, study notes or registry | Records in database, study notes or registry | 66 (20) | 102 (31) | 25 |
| Chang 2004 (22) | WHO definition * | Side effects in last month of treatment | Records in database, study notes or registry | Records in database, study notes or registry | 102 (20) | 14 (3) | 10 |
| Chee 2006 (23) | Treatment interrupted ** | - | Study or clinical notes | Study or clinical notes | 120 (9) | - | - |
| Cherkaoui 2014 (24) | WHO definition * | - | Records in database, study notes or registry | Self-report and study or clinical notes | 91 (33) | - | 13 |
| Chida 2015 (25) | WHO definition * | - | Records in database, study notes or registry | Self-report | ≥21 | - | - |
| Chino 2016 (26) | Discontinuation ** | - | Records in database, study notes or registry | Records in database, study notes or registry | - | 77 (26) | 4 |
| Ciza 2020 (27) | Permanent discontinuation * | Grading using French National Agency of Research on Aids | Records in database, study notes or registry | Records in database, study notes or registry | 3 | 119 | - |
| Colmenero 1997 (28) | Took less than 80% medication ** | Liver function tests | Study or clinical notes | Study or clinical notes | 4 (5) | 7 (8) | 0 |
| Combs 1990 (29) | Therapy interrupted >14 consecutive days ** | - | Study or clinical notes and self-report | Study or clinical notes and self-report | - | - | 71 |
| Dalay 2017 (30) | LFU * | - | Records in database, study notes or registry | Records in database, study notes or registry | 53 (50) | - | 3 |
| Das Shukla 2019 (31) | Interruption * | - | Study or clinical notes | Study or clinical notes | 116 (100) | - | 12 |
| Davtyan 2019 (32) | Not following treatment * | - | Self-report | Self-report | - | - | 45 |
| Deshmukh 2015 (33) | WHO definition * | - | Records in database, study notes or registry | Self-report | 20 (100) | - | 6 |
| Dey 2021 (34) | Therapy no taken ≥28 consecutive days * | - | Study or clinical notes | Self-report | 377 (5) | - | 44 |
| Dhingra 2004 (35) | Temporary interruptions ** | - | Records in database, study notes or registry | Self-report and study or clinical notes | - | 49 (4) | 3 |
| Donald 1998 (36) | Treatment stop ** | Liver function tests | Study or clinical notes | Self-report | - | - | 0 |
| Drobac 2006 (37) | Suspension of therapy for >5 days ** | - | Records in database, study notes or registry | Records in database, study notes or registry | 1 (3) | 16 (42) | 0 |
| Duraisamy 2014 (38) | WHO definition ** | - | Records in database, study notes or registry | Records in database, study notes or registry | 27 (15) | 70 (39) | 1 |
| Elbireer 2011 (39) | WHO definition * | - | Records in database, study notes or registry | Records in database, study notes or registry | - | - | - |
| Fang 2019 (40) | Stopped treatment for ≥1 week * | - | Self-report | Self-report | 262 (100) | - | 61 |
| Ferrer 1991 (41) | Discontinuation * | - | Study or clinical notes | Self-report and study or clinical notes | 20 (11) | - | 6 |
| Franck 2014 (42) | Missing doses * | - | Self-report | Self-report | - | - | - |
| Fry 2005 (43) | Interruption * | - | Study or clinical notes and self-report | Self-report and study or clinical notes | - | - | - |
| Furin 2001 (44) | Discontinuation ** | Liver function tests, clinician discussion | Records in database, study notes or registry | Records in database, study notes or registry | - | 60 (100) | 0 |
| Gafar 2019 (45) | Took <80% medication * | - | Records in database, study notes or registry | Records in database, study notes or registry | 160 (5) | - | 30 |
| Galstyan 2018 (46) | Interruption * | - | - | - | 5 (18) | - | 1 |
| Garazzino 2014 (47) | Treatment stopped ** | - | Study or clinical notes | Study or clinical notes | - | - | 2 |
| Gebremariam 2010 (48) | Default * | - | Study or clinical notes | Self-report | - | - | ≥1 |
| Gonzalez 2012 (49) | Abandonment * | - | Records in database, study notes or registry | Records in database, study notes or registry | 205 (19) | 134 (13) | 8 |
| Gorityala 2015 (50) | Default * | - | Self-report | Self-report | 107 (100) | - | 31 |
| Gualano 2019 (51) | WHO definition * | WHO definition | Records in database, study notes or registry | Records in database, study notes or registry | - | - | 8 |
| Gugssa Boru 2017 (52) | Missed ≥3 doses therapy * | - | Records in database, study notes or registry | Study or clinical notes | 22 (100) | - | 11 |
| Gunawan 2014 (53) | Default * | - | Study or clinical notes | Study or clinical notes | 63 (50) | - | - |
| Gupta 1992 (54) | Took <80% medication * | - | Study or clinical notes and self-report | Study or clinical notes and self-report | - | - | 8 |
| Hamadah 2016 (55) | Discontinuation  * | - | Records in database, study notes or registry | Records in database, study notes or registry | 2 (40) | 5 (100) | 1 |
| Helegbe 2019 (56) | Default * | - | Self-report | Self-report | 20 (21) | - | - |
| Hirpa 2013 (57) | Failure to comply with therapy as prescribed * | - | Records in database, study notes or registry | Self-report; records in database, study notes or registry | Cases: 93 (69) Controls: 10 (7) | - | Cases: 34  Controls: 3 |
| Hoshino 2007 (58) | Noncompliance and discontinuation *** | - | Records in database, study notes or registry | - | 9 (5) | - | 2 |
| Hu 2005 (59) | Non-adherence * | - | Records in database, study notes or registry | Self-report | 400 (47) | - | 103 |
| Isaakidis 2013 (60) | WHO definition * | - | Records in database, study notes or registry | Self-report | 3 (27) | 2 | 1 |
| Jaber 2018 (61) | Default * | - | Study or clinical notes | Study or clinical notes and self-report | 52 (13) | 172 (42) | 42 |
| Jaggarajamma 2007 (62) | WHO definition * | - | Records in database, study notes or registry | Self-report | 186 (20) | - | 59 |
| Jaiswal 2021 (63) | Miss ≥3 doses in a week * | - | Study or clinical notes and self-report | Study or clinical notes and self-report | 55 (100) | - | 20 |
| Janakan 2008 (64) | WHO definition * | - | Study or clinical notes and self-report | Study or clinical notes and self-report | 74 (23) | 26 (8) | 11 |
| Jittimanee 2007 (65) | WHO definition * | - | Study or clinical notes | Study or clinical notes | 54 (34) | - | - |
| Kan 1985 (66) | Discontinuation ** | - | Study or clinical notes | Study or clinical notes | - | 88 (38) | 3 |
| Kandel 2008 (67) | Interruption * | - | Study or clinical notes and self-report | Study or clinical notes and self-report | 58 (26) | - | 4 |
| Keshavjee 2012 (68) | Discontinuation ** | Liver function tests | Records in database, study notes or registry | Records in database, study notes or registry | - | 91 (16) | 0 |
| Khan 2015 (69) | WHO definition * | - | Records in database, study notes or registry | Self-report; records in database, study notes or registry | 4 (33) | - | 2 |
| Kim 2020 (70) | WHO definition *** | - | Study or clinical notes | Records in database, study notes or registry | 780 (100) | - | 387 |
| Kiria 2018 (71) | Discontinuation *** | Severe hepatotoxicity | - | - | 5 (28) | - | 2 |
| Kisambu 2014 (72) | Missed ≥10% of total prescribed therapy * | - | Records in database, study notes or registry | Records in database, study notes or registry | 35 (28) | 65 (52) | 24 |
| Lienhardt 2011 (73) | Drug removal *** | - | Study or clinical notes | Study or clinical notes | - | 67 (6) | 25 |
| Medical Research Council 1989 (74) | Treatment interrupted ** | - | Records in database, study notes or registry | Records in database, study notes or registry | 58 (15) | - | 10 |
| Muture 2011 (75) | WHO definition * | - | Records in database, study notes or registry | Self-report | 945 (48) | - | 13 |
| Nandadeva 2014 (76) | Failed to take treatment >7 days but <2 months * | - | Self-report | Self-report | 30 (100) | - | 5 |
| Orofino 2012 (77) | Discontinuation ** | - | Records in database, study notes or registry | Records in database, study notes or registry | - | - | - |
| Park 2015 (78) | WHO definition * | - | Study or clinical notes | Self-report | 46 (30) | - | 19 |
| Patel 2020 (79) | Non-compliance * | - | Study or clinical notes | Self-report | 31 (31) | - | 23 |
| Piubello 2014 (80) | Stop treatment ** | - | Records in database, study notes or registry | Records in database, study notes or registry | - | 41 (63) | 0 |
| Ranawaka 2021 (81) | Missing or interrupting of therapy consecutively or intermittently ≥4 days per week during intensive phase * | - | Records in database, study notes or registry | Self-report | 46 (18) | 78 (31) | 20 |
| Ranganath 2021 (82) | Taking >90% of doses every month over the last three months * | - | Records in database, study notes or registry; self-report | Records in database, study notes or registry | 125 (25) | - | - |
| Reves 2011 (83) | Discontinuation of treatment * | - | Study or clinical notes | Study or clinical notes | 25 (26) | - | 14 |
| Reyes-Guillen 2008 (84) | Default * | - | Self-report | Self-report | 4 (4) | - | 2 |
| Rodrigues 2010 (85) | Treatment abandonment * | - | Study or clinical notes | Self-report | - | - | - |
| Rupani 2020 (86) | Discontinuation * | - | Self-report | Self-report and study or clinical notes | - | - | 19 |
| Saleem 2015 (87) | Non-compliance * | - | Study or clinical notes | Study or clinical notes | 32 (4) | - | 15 |
| Sanchez-Padilla 2014 (88) | WHO definition and  failure to comply with therapy as prescribed * | - | Self-report | Self-report | 97 (28) | - | ≥11 |
| Santos 2018 (89) | Default * | - | Records in database, study notes or registry | Records in database, study notes or registry | - | - | - |
| Sarpal 2014 (90) | WHO definition * | - | Records in database, study notes or registry | - | 32 (6) | - | 15 |
| Satti 2012 (91) | Treatment suspension ** | - | Records in database, study notes or registry | Study or clinical notes | - | 18 (95) | 1 |
| Setiawan 2019 (92) | Default * | Unexpected effects after receiving anti-TB drugs | Study or clinical notes | Study or clinical notes | - | 172 (100) | 40 |
| Shringarpure 2016 (93) | WHO definition * | - | Records in database, study notes or registry; self-report | Self-report | - | - | - |
| Singh 2017 (94) | WHO definition * | Graded using CTCAE (common terminology criteria for adverse events) | Study or clinical notes | Study or clinical notes | 12 (12) | 39 (39) | 10 |
| Skrahina 2020 (95) | Stop treatment ** | - | Study or clinical notes | - | 2 | - | 0 |
| Snene 2015 (96) | Non-compliance * | - | - | - | - | 48 (89) | - |
| Su 2002 (97) | Discontinuation * | - | Study or clinical notes | Study or clinical notes | 54 (51) | - | 5 |
| Sun 2021 (98) | Discontinuation ** | Defined by Division of Microbiology and Infectious Diseases; determined by TB specialist | Study or clinical notes | Study or clinical notes | ≥11 (≥10) | 30 (26) | 2 |
| Taufiqi 2014 (99) | LFU * | - | Records in database, study notes or registry | Records in database, study notes or registry | - | 19 | 14 |
| Tekle 2002 (100) | Default * | - | Study or clinical notes | Study or clinical notes and self-report | 155 (11) | - | 17 |
| Tesfahuneygn 2015 (101) | <95% treatment taken * | - | Study or clinical notes and self-report | Self-report | - | - | 1 |
| Tsakalidis 1992 (102) | Compliance ** | Temporary intolerance, laboratory tests | Study or clinical notes and self-report | Self-report | 0 | 1 (3) | 0 |
| Ukoha-Kalu 2020 (103) | ≥1 dose missed per month * | - | Study or clinical notes | Study or clinical notes | 44 (73) | - | 11 |
| Veeramani 2015 (104) | WHO definition * | - | Study or clinical notes | Study or clinical notes | 27 (10) | - | 11 |
| Walker 2019 (105) | WHO definition * | Serious ADRs that required a change in therapy | Records in database, study notes or registry | - | 702 (17) | - | - |
| Wares 2003 (106) | >60 days late collecting medication * | - | Records in database, study notes or registry | Study or clinical notes | 30 (49) | - | 11 |
| Wobudeya 2019 (107) | Discontinuation ** | Clinical symptoms and exam | Records in database, study notes or registry | Records in database, study notes or registry | - | >57 (>11) | - |
| Wohlleben 2017 (108) | WHO definition * | - | Study or clinical notes | Study or clinical notes and self-report | 300 (34) | 45 (5) | 26 |
| Woldeyohannes 2021 (109) | LFU * | - | Records in database, study notes or registry | Records in database, study notes or registry | 32 (8) | 40 (10) | 9 |
| Xing 2021 (110) | Interrupted treatment * | - | Study or clinical notes and self-report | Study or clinical notes and self-report | 80 (66) | - | - |
| Xu 2009 (111) | Missed >3 doses * | - | Study or clinical notes | Study or clinical notes | 82 (12) | - | 31 |
| Zhang 1996 (112) | Discontinued treatment ** | - | Study or clinical notes | Study or clinical notes | - | 28 (26) | 1 |
| Zhang 2020 (113) | Missed ≥10% of total prescribed therapy * | - | Self-report | Study or clinical notes | 10 (59) | 8 (47) | 6 |

This table documents how adverse drug reaction (exposure) and missed dose data (outcome) were collected or measured in the included studies. 17 different definitions of missed doses were given. The percentage of the cohort with the outcome and exposure was calculated using the number of participants in the study at the time of the results collection. If this information was not known, the number of participants who were eligible and enrolled in the study was used (Table S6). – (data unknown); * (patient-originated missed doses); ** (HCP-originated missed doses); *** both patient and HCP-originated missed doses); ADR (adverse drug reaction); failure to comply with therapy as prescribed (failure of a patient to comply with or follow the treatment programme and medication regimen as prescribed, including missed single doses); HCP (healthcare professional); LFU (lost/ loss to follow up); records in database, study notes or registry (retrospective information from treatment records, clinical databases and the TB registry); self-report (prospective information collected using a questionnaire, interview or survey); study or clinical notes (prospective study or clinical notes collected by study staff or healthcare professionals in a semi-structured method e.g. directly observed therapy); WHO (World Health Organization); WHO definition (patient who interrupted treatment for at least 2 consecutive months/ 60 days).

**Table S8: Specific adverse drug reactions reported in the context of missed doses**

| **Standardised ADR** | | **DS (81 (65 of which 5*, 10^, 1*^))** | | | | **DR/ MDR/ XDR (66)** | | | | **Mixed (47)** | | | | **Total (178)** |
| --- | --- | --- | --- | --- | --- | --- | --- | --- | --- | --- | --- | --- | --- | --- |
|  |  | **Patient-originated missed doses** | | **HCP-originated missed doses** | | **Patient-originated missed doses** | | **HCP-originated missed doses** | | **Patient-originated missed doses** | | **HCP-originated missed doses** | |  |
| **COS** | **MedDRA terms** | **Evidence of association** | **No evidence of association** | **Evidence of association** | **No evidence of association** | **Evidence of association** | **No evidence of association** | **Evidence of association** | **No evidence of association** | **Evidence of association** | **No evidence of association** | **Evidence of association** | **No evidence of association** |  |
| Blood and Lymphatic System Disorders | Anaemia | 1 (1*^) | 0 | 2 | 1 (1*^) | 0 | 0 | 0 | 0 | 0 | 0 | 0 | 0 | **3** |
|  | Eosinophilia | 0 | 0 | 1 | 0 | 0 | 0 | 0 | 0 | 0 | 0 | 0 | 0 | **1** |
|  | Haematological toxicity | 0 | 0 | 0 | 0 | 0 | 0 | 0 | 0 | 0 | 0 | 1 | 0 | **1** |
|  | Leukopenia | 0 | 0 | 1 | 1 | 0 | 0 | 0 | 0 | 0 | 0 | 0 | 0 | **2** |
|  | Thrombocytopenia | 1 | 0 | 1 | 1 | 0 | 0 | 0 | 0 | 0 | 0 | 0 | 0 | **3** |
| Cardiac Disorders | Bradycardia | 0 | 0 | 0 | 0 | 1 | 0 | 0 | 0 | 0 | 0 | 0 | 0 | **1** |
| Ear and Labyrinth Disorders | Hearing losses | 0 | 0 | 0 | 0 | 0 | 0 | 0 | 3 | 0 | 0 | 0 | 0 | **3** |
|  | Ototoxicity | 0 | 0 | 0 | 0 | 0 | 1 | 0 | 1 | 0 | 0 | 0 | 0 | **2** |
| Endocrine Disorders | Hyperglycaemia | 0 | 0 | 0 | 0 | 0 | 0 | 0 | 1 | 0 | 0 | 0 | 0 | **1** |
|  | Hypothyroidism | 0 | 0 | 0 | 0 | 0 | 0 | 0 | 1 | 0 | 0 | 0 | 0 | **1** |
| Eye Disorders | Eye disorders | 0 | 0 | 0 | 0 | 0 | 0 | 0 | 0 | 1 | 0 | 0 | 0 | **1** |
|  | Optic neuritis | 0 | 0 | 0 | 0 | 0 | 0 | 0 | 2 | 0 | 0 | 0 | 0 | **2** |
|  | Visual acuity reduced | 1 (1^) | 0 | 1 (1^) | 0 | 0 | 0 | 0 | 0 | 0 | 0 | 0 | 0 | **1** |
| Gastrointestinal Disorders | Abdominal discomfort | 1 (1^) | 0 | 1 (1^) | 1 |  | 1 | 0 | 2 | 1 | 0 | 2 | 0 | **8** |
|  | Abdominal pain | 1 | 0 | 1 | 0 | 1 | 0 | 0 | 1 | 1 | 0 | 0 | 0 | **5** |
|  | Constipation | 0 | 0 | 0 | 0 | 1 | 0 | 0 | 0 | 0 | 0 | 0 | 0 | **1** |
|  | Diarrhoea | 1 (1^) | 0 | 1 (1^) | 0 | 0 | 0 | 0 | 0 | 1 | 0 | 0 | 0 | **2** |
|  | Gastritis | 0 | 0 | 0 | 0 | 0 | 0 | 0 | 3 | 1 | 0 | 0 | 0 | **4** |
|  | Gastrointestinal nonspecific dysfunction | 0 | 0 | 0 | 2 | 0 | 0 | 0 | 0 | 0 | 0 | 0 | 0 | **2** |
|  | Nausea and vomiting symptoms | 2 | 1 | 2 | 0 | 2 | 0 | 0 | 2 | 10 | 0 | 0 | 0 | **19** |
|  | Taste disorder | 0 | 0 | 0 | 0 | 1 | 0 | 0 | 0 | 0 | 0 | 0 | 0 | **1** |
| General Disorders and Administration Site Conditions | Asthenia | 2 (1^) | 0 | 1 (1^) | 0 | 0 | 0 | 0 | 0 | 3 | 0 | 0 | 0 | **5** |
|  | Pain | 0 | 1 | 0 | 0 | 2 | 0 | 0 | 0 | 1 | 0 | 0 | 0 | **4** |
| Hepatobiliary Disorders | Acute hepatic failure | 0 | 0 | 1 | 0 | 0 | 0 | 0 | 0 | 0 | 0 | 0 | 0 | **1** |
|  | Elevated liver function test | 0 | 1 | 0 | 2 | 0 | 0 | 1 | 1 | 0 | 0 | 1 | 0 | **6** |
|  | Hepatic cholestatic | 0 | 0 | 0 | 1 | 0 | 0 | 0 | 0 | 0 | 0 | 0 | 0 | **1** |
|  | Hepatic cytolysis | 0 | 0 | 0 | 1 | 0 | 0 | 0 | 0 | 0 | 0 | 0 | 0 | **1** |
|  | Hepatic disorders | 0 | 0 | 0 | 0 | 0 | 0 | 0 | 0 | 1 | 0 | 0 | 0 | **1** |
|  | Hepatitis | 0 | 0 | 2 | 0 | 0 | 0 | 0 | 1 | 1 | 0 | 0 | 0 | **4** |
|  | Hepatotoxicity | 1 (1^) | 0 | 1 (1^) | 1 | 1 | 1 | 1 | 1 | 0 | 0 | 1 | 0 | **7** |
|  | Jaundice | 0 | 0 | 0 | 0 | 0 | 0 | 0 | 1 | 0 | 0 | 0 | 0 | **1** |
|  | Liver injury | 0 | 0 | 0 | 0 | 0 | 0 | 0 | 0 | 0 | 0 | 1 | 0 | **1** |
| Immune System Disorders | Dermatitis allergic | 0 | 0 | 1 (1*) | 1 (1*) | 0 | 0 | 0 | 1 | 0 | 0 | 0 | 0 | **2** |
|  | Hypersensitivity reaction | 1 (1^) | 0 | 1 (1^) | 0 | 0 | 0 | 0 | 0 | 0 | 0 | 1 | 0 | **2** |
| Metabolism and Nutrition Disorders | Decreased appetite | 0 | 0 | 0 | 0 | 1 | 0 | 0 | 0 | 1 | 0 | 0 | 0 | **2** |
|  | Feeding disorder | 0 | 0 | 0 | 0 | 0 | 0 | 0 | 0 | 1 | 0 | 0 | 0 | **1** |
|  | Hyperuricemia | 0 | 0 | 0 | 1 | 0 | 0 | 0 | 0 | 0 | 0 | 0 | 0 | **1** |
| Musculoskeletal and Connective Tissue Disorders | Joint disorders | 2 (1^) | 0 | 1 (1^) | 0 | 0 | 0 | 0 | 4 | 1 | 0 | 0 | 0 | **7** |
|  | Myalgia | 1 | 0 | 0 | 0 | 0 | 0 | 0 | 0 | 0 | 0 | 0 | 0 | **1** |
|  | Myopathy | 1 (1*) | 1 (1*) | 0 | 0 | 0 | 0 | 0 | 0 | 0 | 0 | 0 | 0 | **1** |
| Nervous System Disorders | Dizziness | 1 (1^) | 1 | 1 (1^) | 0 | 1 | 0 | 0 | 0 | 2 | 0 | 0 | 0 | **5** |
|  | Encephalopathies toxic and metabolic | 0 | 0 | 1 | 0 | 0 | 0 | 0 | 0 | 0 | 0 | 0 | 0 | **1** |
|  | Headache | 1 | 0 | 0 | 0 | 1 | 1 | 0 | 1 | 1 | 0 | 0 | 0 | **5** |
|  | Nervous system disorders | 0 | 0 | 0 | 0 | 0 | 1 | 0 | 1 | 0 | 0 | 1 | 0 | **3** |
|  | Paraesthesia | 1 | 0 | 0 | 0 | 0 | 0 | 0 | 0 | 0 | 0 | 0 | 0 | **1** |
|  | Peripheral neuropathy | 2 (1*, 1^) | 1 (1*) | 1 (1^) | 1 | 0 | 0 | 0 | 1 | 0 | 0 | 1 | 0 | **5** |
|  | Syncope | 0 | 1 | 0 | 0 | 0 | 0 | 0 | 0 | 0 | 0 | 0 | 0 | **1** |
|  | Vertigo | 0 | 0 | 0 | 0 | 1 | 0 | 0 | 0 | 0 | 0 | 0 | 0 | **1** |
| Psychiatric Disorders | Anxiety | 0 | 0 | 0 | 0 | 0 | 0 | 0 | 1 | 0 | 0 | 0 | 0 | **1** |
|  | Confusion and disorientation | 1 | 0 | 0 | 0 | 1 | 0 | 0 | 0 | 0 | 0 | 0 | 0 | **2** |
|  | Depression | 0 | 0 | 0 | 0 | 1 | 0 | 0 | 2 | 0 | 0 | 0 | 0 | **3** |
|  | Disturbances in thinking and perception | 0 | 0 | 0 | 0 | 0 | 0 | 0 | 0 | 1 | 0 | 0 | 0 | **1** |
|  | Insomnia | 2 | 0 | 0 | 0 | 0 | 0 | 0 | 0 | 0 | 0 | 0 | 0 | **2** |
|  | Psychiatric disorders | 0 | 0 | 1 | 0 | 1 | 0 | 0 | 2 | 0 | 0 | 0 | 0 | **4** |
|  | Restlessness | 0 | 0 | 0 | 0 | 1 | 0 | 0 | 0 | 0 | 0 | 0 | 0 | **1** |
|  | Sleep disorders | 0 | 0 | 0 | 0 | 0 | 0 | 0 | 0 | 1 | 0 | 0 | 0 | **1** |
|  | Sleep terror | 0 | 0 | 0 | 0 | 0 | 0 | 0 | 0 | 1 | 0 | 0 | 0 | **1** |
|  | Suicidal ideation | 0 | 0 | 0 | 0 | 1 | 0 | 0 | 0 | 0 | 0 | 0 | 0 | **1** |
| Renal and Urinary Disorders | Chromaturia | 0 | 0 | 0 | 0 | 0 | 0 | 0 | 0 | 1 | 0 | 0 | 0 | **1** |
|  | Nephropathy toxic | 0 | 0 | 0 | 0 | 0 | 1 | 0 | 2 | 0 | 0 | 0 | 0 | **3** |
|  | Renal disorders | 0 | 0 | 0 | 0 | 0 | 0 | 0 | 1 | 1 | 0 | 0 | 0 | **2** |
|  | Renal impairment | 0 | 0 | 1 | 0 | 0 | 0 | 0 | 0 | 0 | 0 | 0 | 0 | **1** |
| Skin and Subcutaneous Tissue Disorders | Dermatotoxicity | 0 | 0 | 1 (1*) | 1 (1*) | 0 | 1 | 0 | 1 | 1 | 0 | 1 | 0 | **5** |
|  | Flushing | 0 | 0 | 1 | 0 | 0 | 0 | 0 | 0 | 0 | 0 | 0 | 0 | **1** |
|  | Pallor | 1 | 0 | 0 | 0 | 0 | 0 | 0 | 0 | 0 | 0 | 0 | 0 | **1** |
|  | Pigmentation disorder | 0 | 0 | 0 | 0 | 0 | 0 | 0 | 1 | 0 | 0 | 0 | 0 | **1** |
|  | Pruritus | 3 (1*, 1^) | 0 | 1 (1^) | 1 (1*) | 0 | 0 | 0 | 0 | 3 | 0 | 0 | 0 | **6** |
|  | Rash | 2 | 0 | 1 | 0 | 0 | 0 | 0 | 1 | 1 | 0 | 0 | 0 | **5** |
|  |  | **30 (3*, 10^, 1*^)** | **7 (2*)** | **28 (2*, 10^)** | **16 (3*, 1*^)** | **18** | **7** | **2** | **39** | **37** | **0** | **10** | **0** | **178** |

Evidence from the included studies as to whether reported adverse drug reactions (ADRs), grouped according to their class organ system (COS), were associated with missed doses of treatment. Study populations contained patients with drug-sensitive (DS) disease, drug-resistant (DR)/multidrug-resistant (MDR)/extensively drug-resistant (XDR) disease or populations with mixed drug susceptibility profiles. Studies describing both patient- and HCP-originated missed doses were listed in both categories and distinguished using ^. Studies which showed both mixed evidence for the association between missed doses and ADRs were listed in both categories and distinguished using *. As a result, the total number of ADR occasions appears to be 194 rather than 178 (true total number of occasions). The language used to describe the ADR is standardised using Medical Dictionary for Regulatory Activities Terminology (MedDRA) terminology. Some ADRs were reported at the level of organ system (e.g. ‘haematological disorders’); such descriptors may contain a range of more specific events (e.g. ‘anaemia’). ADRs (adverse drug reactions); DS (drug-sensitive); DR (drug-resistant); HCP (healthcare professional); MDR (multi-drug resistant); MedDRA (Medical Dictionary for Regulatory Activities Terminology); n (number of studies); XDR (extensively drug-resistant).

**References**

1. Linh NN, Viney K, Gegia M, Falzon D, Glaziou P, Floyd K, et al. World Health Organization treatment outcome definitions for tuberculosis: 2021 update. European Respiratory Journal. 2021;58(2):2100804.

2. British National Formulary. Tuberculosis 2022 [Available from: <https://bnf.nice.org.uk/treatment-summaries/tuberculosis/>.

3. World Health Organisation. WHO consolidated guidelines on tuberculosis: drug-susceptible TB treatment 2022 [Available from: <https://www.who.int/publications/i/item/9789240048126>.

4. World Health Organisation. WHO consolidated guidelines on tuberculosis: Drug-resistant TB treatment 2020 [120]. Available from: <https://www.who.int/publications/i/item/9789240007048>.

5. World Health Organisation. WHO announces updated definitions of extensively drug-resistant tuberculosis 2021 [Available from: <https://www.who.int/news/item/27-01-2021-who-announces-updated-definitions-of-extensively-drug-resistant-tuberculosis>.

6. Agudelo CA, Alvarez MF, Hidron A, Villa JP, Echeverri-Toro LM, Ocampo A, et al. Outcomes and complications of hospitalised patients with HIV-TB co-infection. Tropical Medicine & International Health. 2021;26(1):82-8.

7. Akeju OO, Wright SCD, Maja TM. Lived experience of patients on tuberculosis treatment in Tshwane, Gauteng province. Health Sa Gesondheid. 2017;22:259-67.

8. Al-Dossary FS, Ong LT, Correa AG, Starke JR. Treatment of childhood tuberculosis with a six month directly observed regimen of only two weeks of daily therapy. Pediatric Infectious Disease Journal. 2002;21(2):91-7.

9. Ali AOA, Prins MH. Disease and treatment-related factors associated with tuberculosis treatment default in Khartoum State, Sudan: a case-control study. Eastern Mediterranean Health Journal. 2017;23(6):408-14.

10. Amalba A, Bugri AA. Assessing the prevalence and effect of adverse drug reactions among patients receiving first line anti-tubercular medicines in the Tamale Teaching Hospital, Ghana. The Pan African medical journal. 2021;38:191.

11. Amuha MG, Kutyabami P, Kitutu FE, Odoi-Adome R, Kalyango JN. Non-adherence to anti-TB drugs among TB/HIV co-infected patients in Mbarara Hospital Uganda: prevalence and associated factors. African Health Sciences. 2009;9 Suppl 1:S8-15.

12. Auchynka V, Kumar AMV, Hurevich H, Sereda Y, Solodovnikova V, Katovich D, et al. Effectiveness and cardiovascular safety of delamanid-containing regimens in adults with multidrug-resistant or extensively drug-resistant tuberculosis: A nationwide cohort study from Belarus, 2016-18. Monaldi Archives for Chest Disease. 2021;91(1).

13. Barrah S, Jebali H, Khouja N, Khedher R, Fatma LB, Smaoui W, et al. Tuberculosis treatment in dialysis patients. Nephrology Dialysis Transplantation. 2017;32(Supplement 3):iii363.

14. Bartacek A, Schütt D, Panosch B, Borek M. Comparison of a four-drug fixed-dose combination regimen with a single tablet regimen in smear-positive pulmonary tuberculosis. Int J Tuberc Lung Dis. 2009;13(6):760-6.

15. Bastard M, Sanchez-Padilla E, Hewison C, Hayrapetyan A, Khurkhumal S, Varaine F, et al. Effects of treatment interruption patterns on treatment success among patients with multidrug-resistant tuberculosis in Armenia and Abkhazia. Journal of Infectious Diseases. 2015;211(10):1607-15.

16. Burton NT, Forson A, Lurie M, Kudzawu S, Kwarteng E, Kwara A. Factors associated with all-cause mortality among adult patients with tuberculosis attending a ghanaian teaching hospital chest clinic. American Journal of Respiratory and Critical Care Medicine Conference: American Thoracic Society International Conference, ATS. 2011;183(1 MeetingAbstracts).

17. Caceres FD, Orozco LC. Incidence of and factors for non-compliance to antituberculous treatment. Biomedica. 2007;27(4):498-504.

18. Campani STA, Moreira JD, Tietbohel CN. Pulmonary tuberculosis treatment regimen recommended by the Brazilian National Ministry of Health: predictors of treatment noncompliance in the city of Porto Alegre, Brazil. Jornal Brasileiro De Pneumologia. 2011;37(6):776-82.

19. Canete C, Galarza I, Granados A, Farrero E, Estopa R, Manresa F. TUBERCULOUS PLEURAL EFFUSION - EXPERIENCE WITH 6 MONTHS OF TREATMENT WITH ISONIAZID AND RIFAMPICIN. Thorax. 1994;49(11):1160-1.

20. Cardoso MA, Brasil PEAAD, Schmaltz CAS, Sant'Anna FM, Rolla VC. Tuberculosis Treatment Outcomes and Factors Associated with Each of Them in a Cohort Followed Up between 2010 and 2014. BioMed Research International. 2017;2017 (no pagination).

21. Cator M, Brassard P, Ducic S, Culman K. [Factors related to non-compliance with active tuberculosis treatment in Montreal 1992-1995]. Canadian Journal of Public Health Revue Canadienne de Sante Publique. 2002;93(2):92-7.

22. Chang KC, Leung CC, Tam CM. Risk factors for defaulting from anti-tuberculosis treatment under directly observed treatment in Hong Kong. International Journal of Tuberculosis & Lung Disease. 2004;8(12):1492-8.

23. Chee CB, Wang YT, Teleman MD, Boudville IC, Chew SK. Treatment outcome of Singapore residents with pulmonary tuberculosis in the first year after introduction of a computerised treatment surveillance module. Singapore Medical Journal. 2006;47(6):529-33.

24. Cherkaoui I, Sabouni R, Ghali I, Kizub D, Billioux AC, Bennani K, et al. Treatment default amongst patients with tuberculosis in urban Morocco: Predicting and explaining default and post-default sputum smear and drug susceptibility results. PLoS ONE. 2014;9(4) (no pagination).

25. Chida N, Ansari Z, Hussain H, Jaswal M, Symes S, Khan AJ, et al. Determinants of Default from Tuberculosis Treatment among Patients with Drug-Susceptible Tuberculosis in Karachi, Pakistan: A Mixed Methods Study. PLoS ONE [Electronic Resource]. 2015;10(11):e0142384.

26. Chino H, Hagiwara E, Sekine A, Kitamura H, Baba T, Shinohara T, et al. [[Compliance Rate of Standard Treatment Regimen and Optimal Dose of Anti-Tuberculosis Drugs in Late Elderly Patients with Pulmonary Tuberculosis].]. Kekkaku. 2016;91(5):495-502.

27. Ciza F, Gils T, Sawadogo M, Decroo T, Roggi A, Piubello A, et al. Course of adverse events during short treatment regimen in patients with rifampicin-resistant tuberculosis in burundi. Journal of Clinical Medicine. 2020;9(6):1-15.

28. Colmenero JD, Garcia-Ordonez MA, Sebastian MD, Perez-Ruiz E, Sanchez-Lora J, Sanchez-Gonzalez J, et al. [Compliance, efficacy and tolerability of the therapeutic regimen recommended by National Consensus on Tuberculosis]. Enfermedades Infecciosas y Microbiologia Clinica. 1997;15(3):129-33.

29. Combs DL, O'Brien RJ, Geiter LJ. USPHS Tuberculosis Short-Course Chemotherapy Trial 21: effectiveness, toxicity, and acceptability. The report of final results. Annals of Internal Medicine. 1990;112(6):397-406.

30. Dalay V, Yu C, Abong J, Marcelo D. Treatment outcomes of MDR-TB and referral networks in an out-patient private-initiated programmatic management for drug resistant tuberculosis (PMDT) facility. Respirology. 2017;22(Supplement 3):62.

31. Das Shukla A, Chaudhary A. IMPROPER ANTI-TB TREATMENT-ALMOST CERTAIN RECIPE FOR MDR-TB. Journal of Evolution of Medical and Dental Sciences-Jemds. 2019;8(29):2307-10.

32. Davtyan K, Aghabekyan S, Davtyan H, Hayrapetyan A, Aslanyan G. Quality of care provided to tuberculosis patients in Armenia: How satisfied are the patients? Journal of Infection in Developing Countries. 2019;13(5.1):28S-34S.

33. Deshmukh RD, Dhande DJ, Sachdeva KS, Sreenivas A, Kumar AMV, Satyanarayana S, et al. Patient and provider reported reasons for lost to follow up in MDRTB treatment: A qualitative study from a drug resistant TB Centre in India. PLoS ONE. 2015;10(8) (no pagination).

34. Dey A, Lahiri A, Jha SS, Sharma V, Shanmugam P, Chakrabartty AK. Treatment adherence status of the TB patients notified from private sector and its associated factors: Findings of a secondary data analysis from West Bengal, India. Indian Journal of Tuberculosis. 2021.

35. Dhingra VK, Rajpal S, Aggarwal N, Aggarwaln JK, Shadab K, Jain SK. Adverse drug reactions observed during DOTS. Journal of Communicable Diseases. 2004;36(4):251-9.

36. Donald PR, Schoeman JF, Van Zyl LE, De Villiers JN, Pretorius M, Springer P. Intensive short course chemotherapy in the management of tuberculous meningitis. International Journal of Tuberculosis & Lung Disease. 1998;2(9):704-11.

37. Drobac PC, Mukherjee JS, Joseph JK, Mitnick C, Furin JJ, Del Castillo H, et al. Community-based therapy for children with multidrug-resistant tuberculosis. Pediatrics. 2006;117(6):2022-9.

38. Duraisamy K, Mrithyunjayan S, Ghosh S, Nair SA, Balakrishnan S, Subramoniapillai J, et al. Does Alcohol consumption during multidrug-resistant tuberculosis treatment affect outcome?. A population-based study in Kerala, India. Annals of the American Thoracic Society. 2014;11(5):712-8.

39. Elbireer S, Guwatudde D, Mudiope P, Nabbuye-Sekandi J, Manabe YC. Tuberculosis treatment default among HIV-TB co-infected patients in urban Uganda. Tropical Medicine & International Health. 2011;16(8):981-7.

40. Fang XH, Dan YL, Liu J, Jun L, Zhang ZP, Kan XH, et al. Factors influencing completion of treatment among pulmonary tuberculosis patients. Patient Preference and Adherence. 2019;13:491-6.

41. Ferrer X, Kirschbaum A, Toro J, Jadue J, Munoz M, Espinoza A. [Compliance with tuberculosis treatment in adults in Santiago, Chile]. Boletin de la Oficina Sanitaria Panamericana. 1991;111(5):423-31.

42. Franck C, Seddon JA, Hesseling AC, Schaaf HS, Skinner D, Reynolds L. Assessing the impact of multidrug-resistant tuberculosis in children: an exploratory qualitative study. BMC Infectious Diseases. 2014;14:426.

43. Fry RS, Khoshnood K, Vdovichenko E, Granskaya J, Sazhin V, Shpakovskaya L, et al. Barriers to completion of tuberculosis treatment among prisoners and former prisoners in St Petersburg, Russia. International Journal of Tuberculosis and Lung Disease. 2005;9(9):1027-33.

44. Furin JJ, Mitnick CD, Shin SS, Bayona J, Becerra MC, Singler JM, et al. Occurrence of serious adverse effects in patients receiving community-based therapy for multidrug-resistant tuberculosis. International Journal of Tuberculosis & Lung Disease. 2001;5(7):648-55.

45. Gafar F, Van't Boveneind-Vrubleuskaya N, Akkerman OW, Wilffert B, Alffenaar JC. Nationwide analysis of treatment outcomes in children and adolescents routinely treated for tuberculosis in the Netherlands. European Respiratory Journal. 2019;54(6):12.

46. Galstyan A, Borisov SE, Sinitsin M. Bedaquiline in the treatment of patients with co-infection HIV/tuberculosis. European Respiratory Journal Conference: European Respiratory Society International Congress, ERS. 2018;52(Supplement 62).

47. Garazzino S, Scolfaro C, Raffaldi I, Barbui AM, Luccoli L, Tovo PA. Moxifloxacin for the treatment of pulmonary tuberculosis in children: a single center experience. Pediatric Pulmonology. 2014;49(4):372-6.

48. Gebremariam MK, Bjune GA, Frich JC. Barriers and facilitators of adherence to TB treatment in patients on concomitant TB and HIV treatment: a qualitative study. BMC Public Health. 2010;10:651.

49. Gonzalez C, Saenz C, Herrmann E, Jajati M, Kaplan P, Monzon D. [Directly observed treatment for tuberculosis in a Buenos Aires City hospital]. Medicina. 2012;72(5):371-9.

50. Gorityala SB, Mateti UV, Konuru V, Martha S. Assessment of treatment interruption among pulmonary tuberculosis patients: A cross-sectional study. Journal of Pharmacy and Bioallied Sciences. 2015;7(3):226-9.

51. Gualano G, Mencarini P, Musso M, Mosti S, Murachelli S, Cannas A, et al. Putting in Harm to cure: Drug related adverse events do not affect outcome of patients receiving treatment for Multidrug-Resistant Tuberculosis. European Respiratory Journal Conference: 29th International Congress of the European Respiratory Society, ERS Madrid Spain. 2019;54(Supplement 63).

52. Gugssa Boru C, Shimels T, Bilal AI. Factors contributing to non-adherence with treatment among TB patients in Sodo Woreda, Gurage Zone, Southern Ethiopia: A qualitative study. Journal of Infection and Public Health. 2017;10(5):527-33.

53. Gunawan D, Sinaga BYM, Amir Z, Zaluchu F. Factors related to default patients with pulmonary tuberculosis in Medan, Indonesia. Respirology. 2014;3):245.

54. Gupta PR, Gupta ML, Purohit SD, Sharma TN, Bhatnagar M. Influence of prior information of drug toxicity on patient compliance. Journal of the Association of Physicians of India. 1992;40(3):181-3.

55. Hamadah AM, Beaulieu LM, Wilson JW, Aksamit TR, Gregoire JR, Williams AW, et al. Tolerability and Healthcare Utilization in Maintenance Hemodialysis Patients Undergoing Treatment for Tuberculosis-Related Conditions. Nephron. 2016;132(3):198-206.

56. Helegbe GK, Eliasu Y, Aryee PA, Ameade EPK, Sulemana MB, Kuganab-lem R, et al. Factors affecting tb treatment outcomes in the bole district of Ghana: A cross sectional study. Transactions of the Royal Society of Tropical Medicine and Hygiene. 2019;113(Supplement 1):S285-S6.

57. Hirpa S, Medhin G, Girma B, Melese M, Mekonen A, Suarez P, et al. Determinants of multidrug-resistant tuberculosis in patients who underwent first-line treatment in Addis Ababa: a case control study. BMC Public Health. 2013;13:782.

58. Hoshino H, Ohmori M, Yoshiyama T, Wada M, Yamauchi Y, Uchimura K. [New cohort analysis system in new tuberculosis surveillance system in Japan]. Kekkaku. 2007;82(12):897-901.

59. Hu HY, Tsai WC, Kung FT. Factors affecting the failed treatment for tuberculosis patients. [Chinese]. Taiwan Journal of Public Health. 2005;24(4):348-59.

60. Isaakidis P, Paryani R, Khan S, Mansoor H, Manglani M, Valiyakath A, et al. Poor outcomes in a cohort of HIV-infected adolescents undergoing treatment for multidrug-resistant tuberculosis in Mumbai, India. PLoS ONE [Electronic Resource]. 2013;8(7):e68869.

61. Jaber AAS, Khan AH, Sulaiman SAS. Evaluation of tuberculosis defaulters in Yemen from the perspective of health care service. Journal of Pharmaceutical Health Services Research. 2018;9(4):381-92.

62. Jaggarajamma K, Sudha G, Chandrasekaran V, Nirupa C, Thomas A, Santha T, et al. Reasons for non-compliance among patients treated under Revised National Tuberculosis Control Programme (RNTCP), Tiruvallur district, south India. Indian Journal of Tuberculosis. 2007;54(3):130-5.

63. Jaiswal S, Sharma H, Joshi U, Agrawal M, Sheohare R. Non-adherence to anti-tubercular treatment during COVID-19 pandemic in Raipur district Central India. Indian Journal of Tuberculosis. 2021.

64. Janakan N, Seneviratne R. Factors contributing to medication noncompliance of newly diagnosed smear-positive pulmonary tuberculosis patients in the district of Colombo, Sri Lanka. Asia-Pacific Journal of Public Health. 2008;20(3):214-23.

65. Jittimanee SX, Madigan EA, Jittimanee S, Nontasood C. Treatment default among urban tuberculosis patients, Thailand. International Journal of Nursing Practice. 2007;13(6):354-62.

66. Kan GQ, Zhang LX, Wu JC, Ma ZL. Supervised intermittent chemotherapy for pulmonary tuberculosis in a rural area of China. Tubercle. 1985;66(1):1-7.

67. Kandel TR, Mfenyana K, Chandia J, Yogeswaran P. The prevalence of and reasons for interruption of anti-tuberculosis treatment by patients at Mbekweni Health Centre in King Sabata Dalindyebo (KSD) District in the Eastern Cape province. South African Family Practice. 2008;50(6):47-c.

68. Keshavjee S, Gelmanova IY, Shin SS, Mishustin SP, Andreev YG, Atwood S, et al. Hepatotoxicity during treatment for multidrug-resistant tuberculosis: occurrence, management and outcome. International Journal of Tuberculosis & Lung Disease. 2012;16(5):596-603.

69. Khan R, Rafiq M, Ahanger B, Majid A, Jan Y, Rouf ur R. TREATMENT COMPLIANCE OF PATIENTS ON DOTS UNDER RNTCP IN DISTRICT PULWAMA (KASHMIR), WITH SPECIAL REFRENCE TO DEFAULTERS. Journal of Evolution of Medical and Dental Sciences-Jemds. 2015;4(78):13565-9.

70. Kim HW, Min J, Shin AY, Koo HK, Lim SY, Park MJ, et al. Reasons why patients with tuberculosis in South Korea stop anti-TB treatment: a cross-sectional study. International Journal of Tuberculosis & Lung Disease. 2020;24(10):1016-23.

71. Kiria N, Avaliani Z, Bolokadze N, Mikiashvili L. Experience of treatment Pre-XDR and XDR-TB/HIV co-infection with bedaquiline and delamanid containing regimens in Georgia. European Respiratory Journal Conference: European Respiratory Society International Congress, ERS. 2018;52(Supplement 62).

72. Kisambu J, Nuwaha F, Sekandi JN. Adherence to treatment and supervision for tuberculosis in a DOTS programme among pastoralists in Uganda. International Journal of Tuberculosis & Lung Disease. 2014;18(7):799-803.

73. Lienhardt C, Cook SV, Burgos M, Yorke-Edwards V, Rigouts L, Anyo G, et al. Efficacy and safety of a 4-drug fixed-dose combination regimen compared with separate drugs for treatment of pulmonary tuberculosis: the Study C randomized controlled trial. Jama. 2011;305(14):1415-23.

74. Medical Research Council. Management and outcome of chemotherapy for childhood tuberculosis. Medical Research Council Tuberculosis and Chest Diseases Unit. Archives of Disease in Childhood. 1989;64(7):1004-12.

75. Muture BN, Keraka MN, Kimuu PK, Kabiru EW, Ombeka VO, Oguya F. Factors associated with default from treatment among tuberculosis patients in Nairobi province, Kenya: a case control study. BMC Public Health. 2011;11:696.

76. Nandadeva D, Samarabandu WS, Madegedara D. Audit of tuberculosis treatment interrupters in Kandy district, Sri Lanka. Respirology. 2014;3):56.

77. Orofino RD, do Brasil PEA, Trajman A, Schmaltz CAS, Dalcolmo M, Rolla VC. Predictors of tuberculosis treatment outcomes. Jornal Brasileiro De Pneumologia. 2012;38(1):88-97.

78. Park C, Shin H, Lee B, Ban H, Oh I, Kim K, et al. Predictive Factors Associated With Default From Tuberculosis Treatment: A Single Center Case-Control Study In South Korea. American Journal of Respiratory and Critical Care Medicine. 2015;191.

79. Patel AS, Hun V, Adithya S. An exploratory study to identify factors affecting non compliance to dots therapy among TB patients at selected dots centre vadodara. Indian Journal of Public Health Research and Development. 2020;11(3):13-8.

80. Piubello A, Harouna SH, Souleymane MB, Boukary I, Morou S, Daouda M, et al. High cure rate with standardised short-course multidrug-resistant tuberculosis treatment in Niger: no relapses. International Journal of Tuberculosis & Lung Disease. 2014;18(10):1188-94.

81. Ranawaka N, Nandasena S, De Alwis S. Noncompliance of treatment among tuberculosis patients in intensive phase at Kalutara District of Sri Lanka. Indian Journal of Tuberculosis. 2021;68(2):266-71.

82. Ranganath TS, Kishore SG, Reddy R, Murthy HJD, Vanitha B, Sharath BN, et al. Risk factors for non-adherence among people with HIV-associated TB in Karnataka, India: A case-control study. Indian Journal of Tuberculosis. 2021.

83. Reves R, Hamilton CD, Tapy J, Narita M, Kyle RP, Heilig C, et al. Evaluating the efficacy and safety of intermittent tuberculosis (TB) treatment when isoniazid (INH) cannot be used. American Journal of Respiratory and Critical Care Medicine Conference: American Thoracic Society International Conference, ATS. 2011;183(1 MeetingAbstracts).

84. Reyes-Guillen I, Sanchez-Perez HJ, Cruz-Burguete J, Izaurieta-de Juan M. Anti-tuberculosis treatment defaulting: an analysis of perceptions and interactions in Chiapas, Mexico. Salud Publica de Mexico. 2008;50(3):251-7.

85. Rodrigues ILA, Monteiro LL, Pacheco RHB, da Silva SED. Abandonment of tuberculosis treatment among patinets co-infected with TB/HIV. Revista Da Escola De Enfermagem Da Usp. 2010;44(2):380-4.

86. Rupani MB, Dave JD, Parmar VB, Singh MP, Parikh KD. Adverse drug reactions and risk factors for discontinuation of multidrug-resistant tuberculosis regimens in Gujarat, western India. National Medical Journal of India. 2020;33(1):10-4.

87. Saleem U, Mahmood S, Ahmad B. Non compliance to tuberculosis therapy: A cross scetional study. Journal of Applied Pharmacy. 2015;7(2):129-31.

88. Sanchez-Padilla E, Marquer C, Kalon S, Qayyum S, Hayrapetyan A, Varaine F, et al. Reasons for defaulting from drug-resistant tuberculosis treatment in Armenia: a quantitative and qualitative study. International Journal of Tuberculosis & Lung Disease. 2014;18(2):160-7.

89. Santos A, Leung J, Vieira MM, Mello FCQ. Adverse events during drug resistant tuberculosis treatment: The experience of a universitary hospital in Rio De Janeiro, Brazil. American Journal of Respiratory and Critical Care Medicine Conference: American Thoracic Society International Conference, ATS. 2018;197(MeetingAbstracts).

90. Sarpal SS, Goel NK, Kumar D, Janmeja AK. Reasons for interruption of anti-tubercular treatment among the retreatment patients in category II of RNTCP in Chandigarh, north India. Indian Journal of Tuberculosis. 2014;61(2):121-8.

91. Satti H, McLaughlin MM, Omotayo DB, Keshavjee S, Becerra MC, Mukherjee JS, et al. Outcomes of comprehensive care for children empirically treated for multidrug-resistant tuberculosis in a setting of high HIV prevalence. PLoS ONE [Electronic Resource]. 2012;7(5):e37114.

92. Setiawan SI, Ascobat P. Adverse reactions to first-line anti-tuberculosis drugs as a risk factor of pulmonary tuberculosis treatment default in Jakarta, Indonesia. International Journal of Applied Pharmaceutics. 2019;11(Special Issue 6):80-3.

93. Shringarpure KS, Isaakidis P, Sagili KD, Baxi RK, Das M, Daftary A. "When Treatment Is More Challenging than the Disease": A Qualitative Study of MDR-TB Patient Retention. PLoS ONE [Electronic Resource]. 2016;11(3):e0150849.

94. Singh A, Sayedda K, Ahmed QS. Comparison of adverse drug reactions of antitubercular drugs in category 1 tuberculosis patients between daily and intermittent regimen and its impact on outcome. Journal of Research in Medical and Dental Science. 2017;5(1):6-12.

95. Skrahina A, Yatskevich N, Hurevich H, Grankov V, Klimuk D, Solodovnikova V, et al. Treatment of multidrug-resistant tuberculosis (MDR-TB) with modified shorter all-oral treatment regimen (mSTR) under operational research conditions in Belarus. European Respiratory Journal Conference: European Respiratory Society International Congress, ERS. 2020;56(Supplement 64).

96. Snene H, Daghfous H, Saad SB, Khelifa MB, Belhadj S, Slim L, et al. Could side effects of MDR-TB treatment change the outcome of the disease? European Respiratory Journal Conference: European Respiratory Society Annual Congress. 2015;46(SUPPL. 59).

97. Su WJ, Perng RP. Fixed-dose combination chemotherapy (Rifater/Rifinah) for active pulmonary tuberculosis in Taiwan: a two-year follow-up. Int J Tuberc Lung Dis. 2002;6(11):1029-32.

98. Sun W, Wu Z, Zhou Y, Xia F, Tang Q, Wang J, et al. A highly effective and inexpensive standardized treatment of multidrug-resistant tuberculosis: a multicenter prospective study in China. BMC Infectious Diseases. 2021;21(1):834.

99. Taufiqi HRA, Harsini, Aphridasari J. Common causes of loss to follow up of multidrug resistant tuberculosis patients in moewardi hospital Surakarta 2011-2013. Respirology. 2014;3):239.

100. Tekle B, Mariam DH, Ali A. Defaulting from DOTS and its determinants in three districts of Arsi Zone in Ethiopia. International Journal of Tuberculosis & Lung Disease. 2002;6(7):573-9.

101. Tesfahuneygn G, Medhin G, Legesse M. Adherence to Anti-tuberculosis treatment and treatment outcomes among tuberculosis patients in Alamata District, northeast Ethiopia. BMC Research Notes. 2015;8:503.

102. Tsakalidis D, Pratsidou P, Hitoglou-Makedou A, Tzouvelekis G, Sofroniadis I. Intensive short course chemotherapy for treatment of Greek children with tuberculosis. Pediatric Infectious Disease Journal. 1992;11(12):1036-42.

103. Ukoha-Kalu BO, Adibe MO. Determinants of medication adherence among patients on concomitant tuberculosis and antiretroviral therapy in Kogi State Nigeria. International Journal of Pharmacy Research and Technology. 2020;10(2):34-9.

104. Veeramani G, Madhusudhan S. Study on default among tuberculosis patients treated under directly observed treatment short course. Der Pharmacia Lettre. 2015;7(12):163-8.

105. Walker IF, Shi O, Hicks JP, Elsey H, Wei X, Menzies D, et al. Analysis of loss to follow-up in 4099 multidrug-resistant pulmonary tuberculosis patients. European Respiratory Journal. 2019;54(1) (no pagination).

106. Wares DF, Singh S, Acharya AK, Dangi R. Non-adherence to tuberculosis treatment in the eastern Tarai of Nepal. International Journal of Tuberculosis & Lung Disease. 2003;7(4):327-35.

107. Wobudeya E, Jaganath D, Sekadde MP, Nsangi B, Haq H, Cattamanchi A. Outcomes of empiric treatment for pediatric tuberculosis, Kampala, Uganda, 2010-2015. BMC Public Health. 2019;19(1):446.

108. Wohlleben J, Makhmudova M, Saidova F, Azamova S, Mergenthaler C, Verver S. Risk factors associated with loss to follow-up from tuberculosis treatment in Tajikistan: a case-control study. BMC Infectious Diseases. 2017;17(1):543.

109. Woldeyohannes D, Tekalegn Y, Sahiledengle B, Assefa T, Aman R, Hailemariam Z, et al. Predictors of mortality and loss to follow-up among drug resistant tuberculosis patients in Oromia Hospitals, Ethiopia: A retrospective follow-up study. PLoS ONE [Electronic Resource]. 2021;16(5):e0250804.

110. Xing W, Zhang R, Jiang W, Zhang T, Pender M, Zhou J, et al. Adherence to multidrug resistant tuberculosis treatment and case management in chongqing, china - a mixed method research study. Infection and Drug Resistance. 2021;14:999-1012.

111. Xu WG, Lu W, Zhou Y, Zhu LM, Shen HB, Wang JM. Adherence to anti-tuberculosis treatment among pulmonary tuberculosis patients: a qualitative and quantitative study. Bmc Health Services Research. 2009;9.

112. Zhang L-X, Kan G-Q, Tu D-H, Wan L-Y, Faruqi AR. Fixed-dose combination chemotherapy versus multiple, single-drug chemotherapy for tuberculosis. Current Therapeutic Research. 1996;57(11):849-56.

113. Zhang J, Yang Y, Qiao X, Wang L, Bai J, Yangchen T, et al. Factors influencing medication nonadherence to pulmonary tuberculosis treatment in tibet, china: A qualitative study from the patient perspective. Patient Preference and Adherence. 2020;14:1149-58.
